# Oncogenic function of the homeobox A13-long noncoding RNA HOTTIP-insulin growth factor-binding protein 3 axis in human gastric cancer

**DOI:** 10.18632/oncotarget.9102

**Published:** 2016-04-29

**Authors:** Sophie S.W. Wang, Kenly Wuputra, Chung-Jung Liu, Yin-Chu Lin, Yi-Ting Chen, Chee-Yin Chai, Chen-Lung Steve Lin, Kung-Kai Kuo, Ming-Ho Tsai, Shin-Wei Wang, Ker-Kong Chen, Hiroyuki Miyoshi, Yukio Nakamura, Shigeo Saito, Tadashi Hanafusa, Deng-Chyang Wu, Chang-Shen Lin, Kazunari K. Yokoyama

**Affiliations:** ^1^ Division of Gastroenterology, Department of Internal Medicine, Kaohsiung Medical University Hospital, Kaohsiung 807, Taiwan; ^2^ Center for Stem Cell Research, Kaohsiung Medical University Hospital, Kaohsiung 807, Taiwan; ^3^ Graduate Institute of Medicine, Kaohsiung Medical University, Kaohsiung 807, Taiwan; ^4^ School of Dentistry, Kaohsiung Medical University, Kaohsiung 807, Taiwan; ^5^ Department of Pathology, Kaohsiung Medical University Hospital, Kaohsiung 807, Taiwan; ^6^ Department of Surgery, Kaohsiung Medical University Hospital, Kaohsiung 807, Taiwan; ^7^ Department of Physiology, Keio University School of Medicine, Shinanomachi, Tokyo 160-8582, Japan; ^8^ Cell Engineering Division, BioResource Center, RIKEN, Tsukuba, Ibaraki 305-0074, Japan; ^9^ School of Science and Engineering, Teikyo University, Utsunomia, Tochigi 329-2192, Japan; ^10^ Saito Laboratory of Cell Technology, Yaita, Tochigi 329-2192, Japan; ^11^ Advanced Science Research Center, Okayama University, Okayama, Okayama 700-8558, Japan; ^12^ Department of Medicine, College of Medicine, Kaohsiung Medical University, Kaohsiung 807, Taiwan; ^13^ Department of Internal Medicine, Kaohsiung Municipal Ta-Tung Hospital, Kaohsiung 807, Taiwan; ^14^ Center of Infectious Disease and Cancer Research, Kaohsiung Medical University, Kaohsiung 807, Taiwan; ^15^ Department of Biological Sciences, National Sun Yat-sen University, Kaohsiung 804, Taiwan; ^16^ Center of Environmental Medicine, Kaohsiung Medical University, Kaohsiung 807, Taiwan; ^17^ Faculty of Science and Engineering, Tokushima Bunri University, Sanuki 763-2193, Japan; ^18^ Department of Molecular Preventive Medicine, Graduate School of medicine, The University of Tokyo, Tokyo 113-0033, Japan

**Keywords:** gastric cancer cells, HOTTIP, HoxA13, IGFBP-3, p53-E2F signaling

## Abstract

To study the mechanisms of gastric tumorigenesis, we have established CSN cell line from human normal gastric mucosa, and CS12, a tumorigenic and invasive gastric cancer cell line from CSN passages. Many stem cell markers were expressed in both CSN and CS12 cells, but LGR5 and NANOG were expressed only in CS12 cells. Increased expression of homeobox A13 (HoxA13) and its downstream cascades was significant for the tumorigenic activity of CS12 cells, and was associated with recruitment of E2F-1 to *HoxA13* promoter accompanied with increased trimethylation of histone H3 lysine 4 (H3K4me3) at the hypomethylated E2F motifs. Knockdown of *HoxA13* caused the downregulation of long non-coding RNA HOTTIP and insulin growth factor-binding protein 3 (IGFBP-3) genes, indicating that both were targets of HoxA13. Concurrent regulation of HoxA13-HOTTIP was mediated by the mixed lineage leukemia-WD repeat domain 5 complex, which caused the trimethylation of H3K4 and then stimulated cell proliferation. HoxA13 transactivated the *IGFBP-3* promoter through the HOX-binding site. Activation of IGFBP-3 stimulated the oncogenic potential and invasion activity. Increased expression of HoxA13 (63.2%) and IGFBP-3 (28.6%) was detected in human gastric cancer tissues and was found in the gastric cancer data of The Cancer Genome Atlas. Taken together, the HoxA13–HOTTIP–IGFBP-3 cascade is critical for the carcinogenic characteristics of CS12 cells.

## INTRODUCTION

Homeobox genes are known as the transcriptional regulators of mammalian embryogenic development and are deregulated in tumorigenesis. There are few known direct targets of Hox proteins, and their mechanism of regulation is incompletely understood. The homeobox A13 (HoxA13) gene is the most posterior of the *HOX* clusters in 7p15.2. This gene is expressed in the genital tubercle during embryogenesis [1, 2] and plays an essential role in skeletogenesis, interdigital programmed cell death, and cell sorting of autopod formation. The loss of HoxA13 function in mice causes missing phalanx elements and affects the carpal and tarsal regions [3]. In humans, mutations in HoxA13 are associated with dominantly inherited hand–foot–genital syndrome (HFGS; OMIM #140000) [4, 5] and Guttmacher syndrome (GS; OMIM #176305), which include limb and genitourinary abnormalities [6, 7]. Similar malformations have also been observed in the spontaneous mouse mutants, hypodactyly [8], and in engineered *HoxA13* -null mouse models [9, 10]. HoxA13 is essential for placental vascular patterning and labyrinth endothelial specification through direct regulation of tyrosine kinase with immunoglobulin-like and epidermal growth factor-like domain 1 and forkhead box F1 [11].

The role of HoxA13 in cancer progression has been reported in hepatocarcinogenesis [12], especially in the liver stem-like cell lines [13], and in prostatic neoplasia [14], leukemogenesis [15], and esophageal squamous cell carcinoma [16]. HoxA13 is a prognostic marker of the aggressive phenotype of gastric cancer [17]. However, the mechanism underlying HoxA13-mediated gastric carcinogenesis and progression of gastric cancer is unclear.

Long noncoding RNAs (lncRNAs) that do not encode proteins are defined as transcripts containing > 200 nucleotides. lncRNAs account for more than 90% of the transcriptome and are typically transcribed by RNA polymerase II. They play an essential role in the control of gene expression involved in various physiological processes, including development, differentiation, and metabolism [18]. HOTTIP lncRNA is located at the 5′-end of the HoxA cluster and is associated with the polycomb repressive complex 2 (PRC2) and WD repeat domain 5 (WDR5) [19]. The interaction between HOTTIP and the WDR5–mixed lineage leukemia (MLL) complex increases histone H3 lysine 4 trimethylation and activates the expression of multiple 5′-HoxA genes [19]. Recent reports have shown that HOTTIP is associated with cancer metastasis and is a negative prognostic factor in patients with liver and tongue cancer [20, 21]. In addition, HOTTIP expression promotes cancer progression and drug resistance by regulating HoxA13 in pancreatic cancer [22]. Another study shows that HOTTIP increases pancreatic cancer cell proliferation, survival, and migration through HoxA family genes other than HoxA13 [23].

The insulin-like growth factor-binding protein-3 (IGFBP-3) influences several molecular mechanisms or signaling pathways that determine cell death or survival, particularly in the context of cancer. Whereas the biological activity of IGFBP-3 is attributed in part to its ability to bind and neutralize insulin-like growth factors (IGF), thereby inhibiting IGF receptor (IGFR) activation, there is other evidence that IGFBP-3 also has intrinsic IGF- or IGF1R-independent effects that influence cell fate. IGFBP-3 inhibits cell growth and apoptosis in some circumstances but stimulates cell growth and survival in others [24–26]. IGFBP-3 is known to bind nuclear receptors of retinoic acid, vitamin D, peroxisome proliferator-activated receptor γ, nuclear hormone receptor 77, and epidermal growth factor receptors as well as the protein kinase catalytic subunits of DNA repair enzymes [25]. IGFBP-3 is known as a transcriptional target of the tumor suppressor protein p53, which modulates IGFBP-3 [26, 27]. However, the relationship between HoxA13 and IGFBP-3 remains elusive.

The progression of gastric cancer is recognized as a multistep process that involves the activation of oncogenes and inactivation of tumor suppressor genes [28, 29]. We have previously established a nonmalignant gastric cell line, CSN, from the stomach mucosa of a patient with mild gastritis, which exhibits features of stem/progenitor cells [30]. After a prolong expansion of CSN cells, a tumorigenic subline CS12 was generated, which exhibited anchorage-independent growth, xenograft tumor formation in nude mice, duplication of the short arm of chromosome 7 (7p15.1–15.3 and 7p22.1–22.3) on chromosome 12, and increased expression of HoxA cluster genes when compared with the nontumorigenic CSN cells [31]. Thus, the increased expression of HoxA genes may contribute to gastric tumorigenesis. Here, we examined the role of HoxA13 in contributing to the cancerous characteristics of CS12 cells and identified the HoxA13-HOTTIP-IGFBP-3 axis as the underlying mechanism.

## RESULTS

### CS12 cells exhibited more aggressive cancerous features than CSN cells

**Figure 1:**
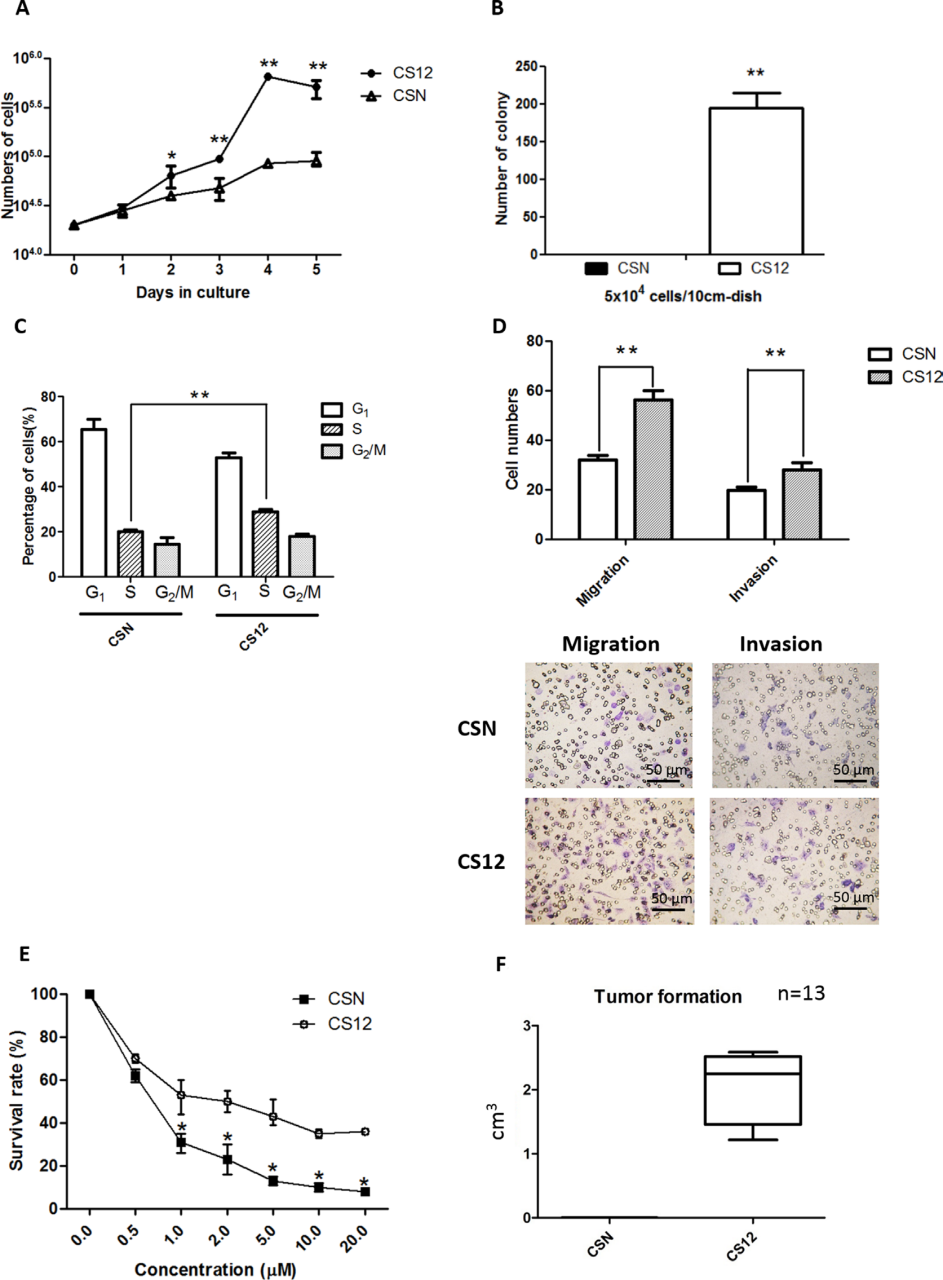
Comparative features of CSN and CS12 cells (**A**) Cell proliferation of CSN and CS12 cells. The mean number of cells (trypan blue dye-exclusion test) was determined for five independent plates. CSN and CS12 cells were starved in MEMα containing 0.1% FCS for 24 h and then replated in MEMα containing 10% FCS and cultured for 5 days. (**B**) Colony formation of CSN and CS12 cells. Cells were plated in gelatin-coated dishes and colonies with a diameter > 2 mm were counted 2 weeks later. (**C**) Serum-starved cells were cultured in DMEM containing 10% FCS for 18 h, stained with PI, and subjected to flow cytometric analysis to determine the percentage of cells in each cell cycle. (**D**) The percentage of invasion and migration was calculated based on the ratio of the number of invading cells vs the total number of CSN and CS12 cells used in inoculation. (**E**) The drug resistance capacity of CS12 cells was measured as the survival rate of cells when exposed to drug such as 5FU. (**F**) Tumor sizes with time in SCID mice subcutaneously injected with CS12 cells and CSN normal cells. CS12 cells, not CSN cells, form tumors. Data in A–E were derived from five independent experiments and are presented as mean ± SEM (two-tailed Student *t* test; *p < 0.05; **p < 0.01).

To characterize the cancerous features of CS12 cells *in vitro*, cell growth, colony formation, cell motility, and chemoresistance between CSN and CS12 cells, were compared. A trypan blue dye exclusion assay showed that CS12 cells proliferated more rapidly than CSN cells (Figure 1A). A colony-formation assay showed that CS12 (5 × 10^4^ cells) generated about 200 colonies, but CSN cells did not produced any colonies (Figure 1B). Cell-cycle analysis showed that 27%–32% of CS12 cells were in S-phase, whereas only 18%–22% of CSN cells were in S-phase (Figure 1C). All data were consistent with the more proliferative nature of CS12 cells. A Transwell invasion assay demonstrated that both the invasion and the migration efficiencies were 1.4-fold higher in CS12 cells than in CSN cells (Figure 1D), which showed that CS12 exhibited increased migration and invasion activities. These findings were supported by the elevated expression of matrix metalloproteinase (MMP) and epithelial to mesenchymal transition (EMT) genes, such as *Snail* and *Zeb* 1 in CS12, but not the expression of *Twist* (Supplementary Figure 1A). Their sensitivity to 5-fluorouracil, a common anticancer drug used for treatment of gastric cancer [32], was examined. The results showed that CS12 were more resistant to 5-fluorouracil than CSN cells (Figure 1E). Their *in vivo* tumorigenicity was examined using a xenograft transplantation test and only CS12 cells generated tumors in SCID mice (Figure 1F) consistent with previous findings [30, 31]. The tumor showed 10% malignant cells that contained little cytoplasm (Supplementary Figure 1B). Taken together, these data indicate that CS12 cells exhibit more cancerous characteristics than CSN cells.

### Differential expression of stemness genes and pluripotency in CSN and CS12 cells

Acquired chemoresistance and EMT are well-known as hallmarks of cancer stem cell-like cells [32, 33]. Both CS12 and CSN cells express OCT4 stemness genes [30, 31]. Here, we further characterized whether CS12 possessed typical stem cell features. Immunofluorescence analysis showed the expression of stemness markers including OCT4, SOX2, KLF4, SSEA-3, SSEA-4, TRA-1-60, and TRA-1-81 in both CSN and CS12 cells, but LGR5 and NANOG were only detected in CS12 cells (Supplementary Figure 2A, 2B and 2C). Semiquantitative reverse transcription-polymerase chain reaction (RT-PCR) analysis showed that the transcript levels of SOX2, NANOG, hTERT, and REX1 were significantly higher in CS12 compared with CSN cells (Supplementary Figure 2D). These data suggest that CS12 cells exhibit more stem-like cell characteristics.

We next examined the pluripotency of CS12 cells by inducing teratoma formation. CS12 differentiated into cells including osteoblasts, muscle cells, and megakaryocytes that were derived from two germ layers (Supplementary Figure 2E). Neither CSN nor CS12 cells stained positively for alkaline phosphatase (data not shown), suggesting that CSN and CS12 cells were not pluripotent stem cells.

### Upregulation of HoxA13 was critical for the tumorigenic properties of CS12 cells

**Figure 2:**
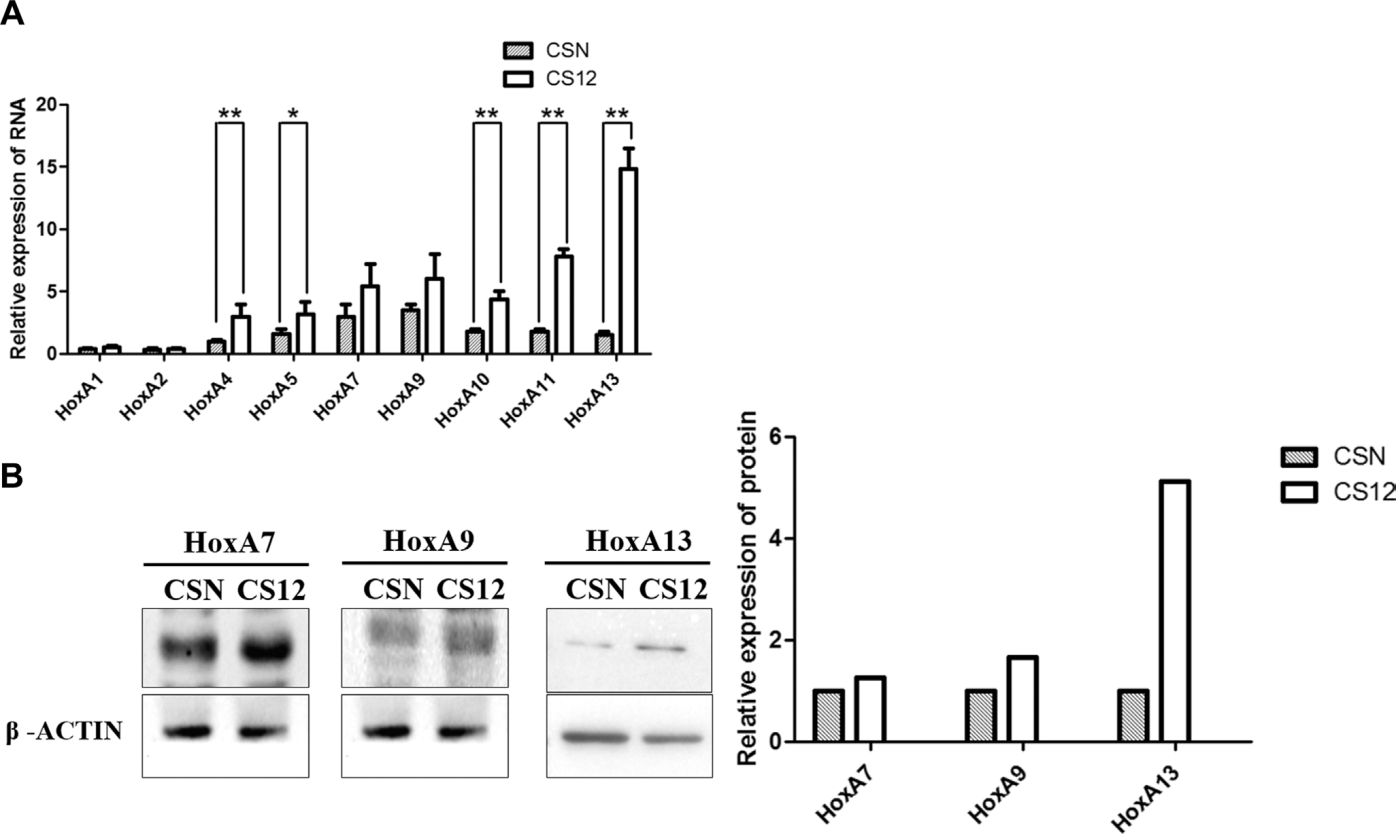
Comparative expression of HOXA family in CSN and CS12 cells (**A**) Comparative mRNA expression of *HOXA* family genes in CSN and CS12 cells was examined by semiquantitative RT-PCR as shown in the Materials and Methods. The data were derived from five independent experiments, and are presented as mean ± SEM (two-tailed Student's *t* test; **p < 0.01). Relative expression was calculated by normalization of the HoxA4 mRNA in CSN cells as 1.0. (**B**) Comparative expression of HoxA7, HoxA9 and HoxA13 proteins was examined by western blot in CSN and CS12 cells. The intensity of bands in western blotting was quantitated using GeneTools (Syngene USA, Frederick, MD, USA) and Image Lab software (Bio-Rad). The relative intensity of each band was calculated by normalization of the corresponding band image of CSN as 1.0.

We previously reported that CS12 cells exhibited duplicated chromosome 7 short arm where HoxA genes reside [4]. Both the qPCR and western blot data showed that HoxA13 was 4.7- to 15-fold upregulated in CS12 than in CSN cells (Figure 2A and 2B). These results suggested that HoxA13 was upregulated through mechanisms other than simply duplicated gene dosage.

**Figure 3:**
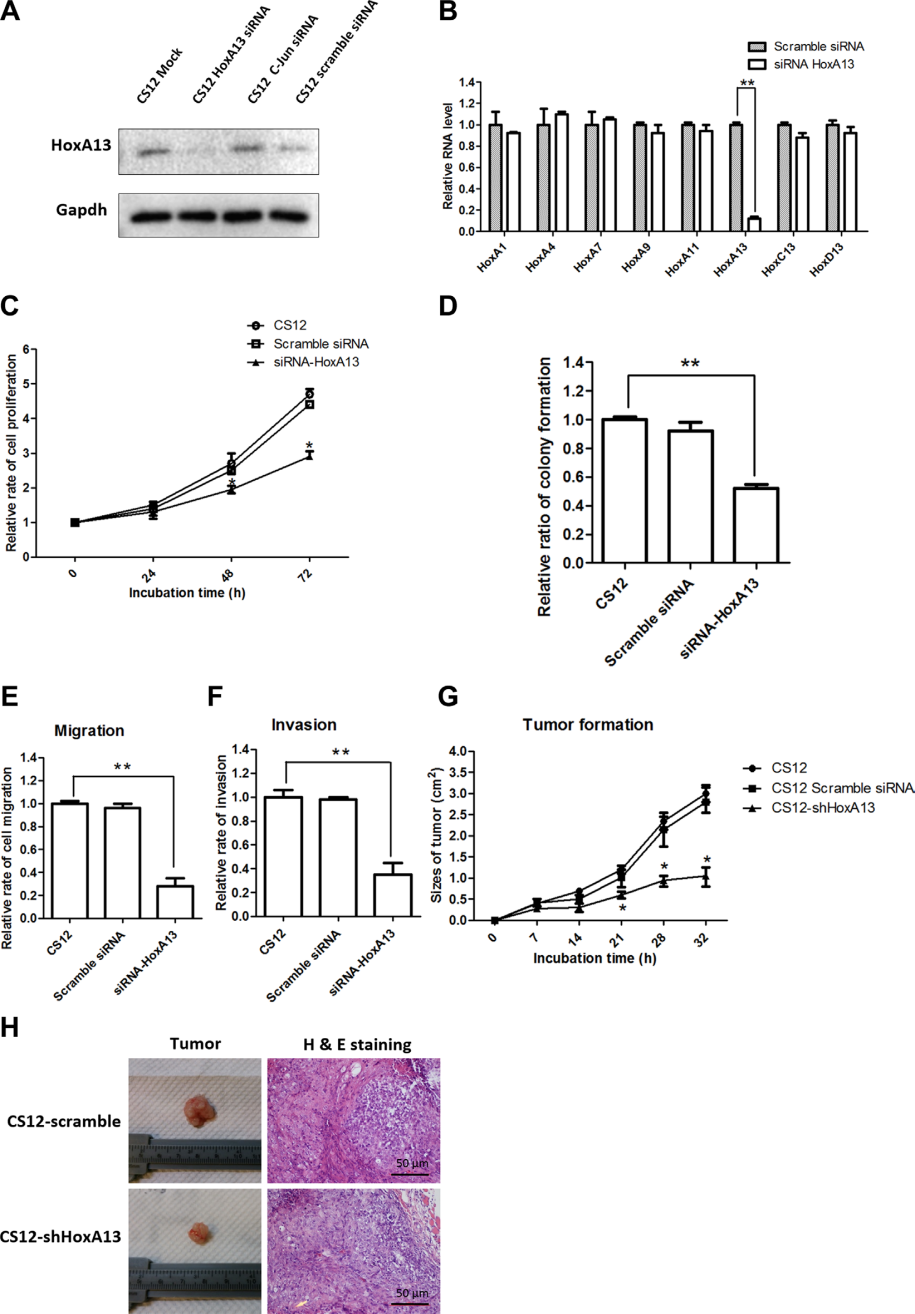
Effect of *HoxA13* knockdown in tumorigenicity of CS12 cells (**A**) Expression of HoxA13 was examined by western blot in CS12 cells treated with siRNA against *HoxA13, c-Jun, IGFBP-3*, or scrambled control as described in the Materials and Methods. (**B**) Expression of mRNA levels of the HOX family was examined by qPCR as described in the Materials and Methods. The level of HoxA1 mRNA in scramble siRNA treated CS12 cells was considered to be 1.0. (**C**) The effects of siRNA-HoxA13 on cell growth in CS12 cells were assessed as described in the Materials and Methods. (**D**) The effects of siRNA-HoxA13 on colony formation in CS12 cells was assessed as described in Figure 1B. The effects of siRNA-HoxA13 on migration (**E**) and invasion (**F**) activities were assessed as described in Figure 1D. (**G**) The effects of the siRNA-HoxA13 on tumor formation. The siRNA-HoxA13 and scramble RNA were introduced into CS12 cells (5 × 10^6^), and then the cells were injected subcutaneously into male SCID mice as described in the Materials and Methods, and the tumor size was measured. (**H**) Representative image of the tumor. All data in B–G were derived from six independent experiments and are presented as mean ± SEM (two-tailed Student *t* test; *p < 0.05, **p < 0.01).

Because of the oncogenic role of HoxA13, we suspect that high expression of HoxA13 may contribute to gastric tumorigenesis of CS12 from CSN cells. To test this hypothesis, HoxA13 expression was knocked down in CS12 cells and then the cell growth, cell mobility, colony formation, and tumor formation in SCID mice were examined. Knockdown by *HoxA13* siRNA significantly reduced HoxA13 expression at protein (Figure 3A) and mRNA (Figure 3B) levels, but the scrambled siRNA and off-target *C-Jun* siRNA did not affect the expression of HoxA13. After knockdown of HoxA13 expression in CS12 cells, the cell proliferation activity (Figure 3C) and colony formation ability (Figure 3D) were reduced by about 50%. The migration and invasion activities of HoxA13-knockdown CS12 cells were decreased to around 30% of controls (Figure 3E and 3F). Knockdown of HoxA13 expression induced by shHoxA13 lentivirus also significantly impaired tumor formation ability of CS12 cells in SCID mice (Figure 3G and 3H). Hematoxylin and eosin staining of tumor sections demonstrated that tumor regions shrank by knockdown of *HOXA13* (Figure 3H). These results indicate that elevated *HoxA13* expression is critical for the cancerous features of CS12 cells.

### Hypomethylation of the *HoxA13* promoter at the p53/E2F-binding site in CS 12 cells

**Figure 4:**
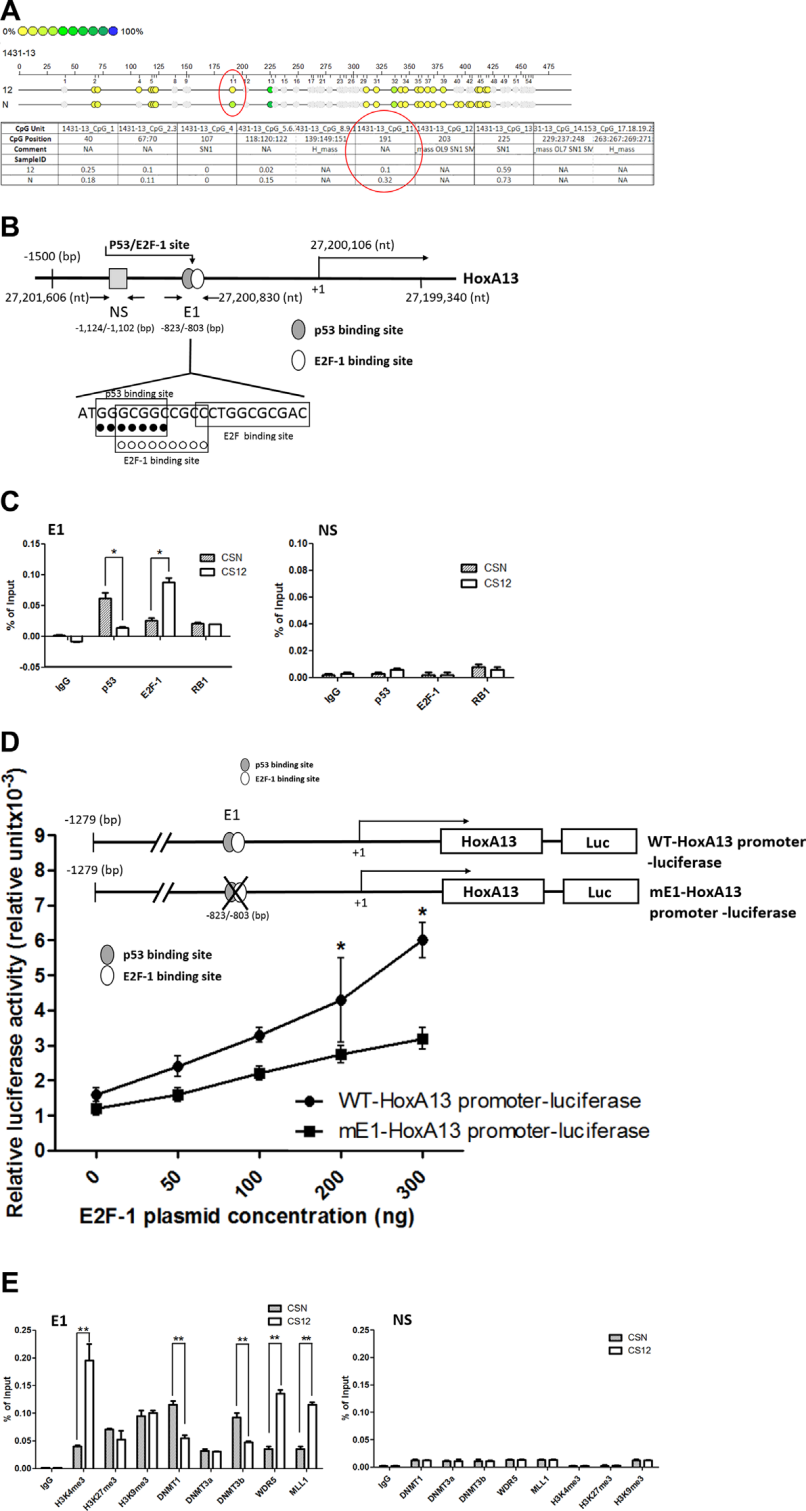
DNA methylation of E1 site in CpG islands of *HoxA13* promoter (**A**) DNA methylation analysis of *HoxA13* promoter. The relative extent of DNA methylation is indicated as intensity; complete methylation with a value of 1.0 is shown as green and hypomethylation is indicated by yellow dots. The red circle shows significant difference in DNA methylation at CpG 11 (1,431–13 segment) of the *HoxA13* promoter. N: CSN cells; 12; CS12 cells. NA indicates not added. (**B**) Schematic representation of the promoter region of *HoxA13* gene. The differential methylated CpG 11 site was found to be the p53/E2F-1 binding site (E1 site; −823 to −803 bp). A nonspecific site (NS; gray square) for ChIP-qPCR was assigned at −1,124 to −1,102 (bp). +1 indicates the transcriptional start site. (**C**) ChIP–qPCR analysis of p53, E2F-1, RB-1, and IgG (negative control) was performed in CSN and CS12 cells as described in the Materials and Methods. Input DNA (1/20-fold) was also analyzed. (**D**) Luciferase-linked wild type (WT) or mE1 mutant-promoter, control pGL4, and various amounts (0, 50, 100, 200, and 300 ng) of pCMV-SPORT6-E2F-1 were transfected into CS12 cells, and luciferase activity was measured as described in the Materials and Methods. (**E**) ChIP–qPCR analysis using antibodies against DNA methyltransferase family members, methylated histones, and the WDR5–MLL complex were performed in CSN and CS12 cells. Input DNA (1/20-fold) was also analyzed. The data C-E are presented as mean ± SEM (two-tailed Student *t* test; *p < 0.05, **p < 0.01).

To investigate the mechanism underlying HoxA13 overexpression in CS12 cells, we first examined the DNA methylation of *HoxA13* promoter by sodium bisulfite conversion followed by pyrosequencing analysis. The results showed a decreased DNA methylation (ratio; 0.1 vs 0.32) at the CpG position 191 of *HoxA13* promoter in CS12 when compared with CSN cells (Figure 4A). Notably, the CpG position 191 is a composite p53/E2F-binding site (27,200,830; designated as the E1 site) (Figure 4B). To examine the differential binding of p53 and E2F1 at this position in CS12 and CSN cells, chromatin immunoprecipitation (ChIP) assays were conducted. The results showed that recruitment of p53 to the E1 site of *HoxA13* promoter was decreased in CS12 cells, whereas the interaction between E2F1 and E1 site was increased (Figure 4C). The recruitment of RB1 was similar in CSN and CS12 cells and the nonspecific (NS) site in the *HOXA13* promoter did not recruit p53 or E2F1. Thus, the recruitment of E2F-1 to the E1 site is critical for the activation of *HoxA13* promoter in CS12 cells. In attempt to confirm this observation further, we performed the forced expression of E2F-1 to observe the enhanced expression of *HoxA13* promoter. We generated the *HoxA13* promoter-luciferase constructs of wild type- (WT-) and its E1 mutant (mE1)-promoter luciferase and examined the effects of E2F-1. The expression of WT- *HoxA13* promoter was more greatly increased by overexpression of E2F-1 than that of mE1 mutant promoter in CS12 cells (Figure 4D) and CSN cells (Supplementary Figure 3A). Thus, a decrease of p53 binding at the E1 site by ChIP assay was consistent with the reduction of p53 and p21 expression in CS12 cells (Supplementary Figure 4A and 4B). By contrast, further addition of *p53* significantly repressed the expression of WT- *HoxA13* promoter in CSN cells (Supplementary Figure 3B) and the transactivation activity of p53 and the p53-regulated *p21^Cip1^* promoter activity were also decreased in CS12 when compared with those in CSN cells (Supplementary Figure 4C and 4D). These results suggest that expression of E2F-1 was increased and expression of p53 was decreased in CS12 cells as compared with CSN cells. These results were consistent with the increase of S phase in CS12 compared with CSN cells (Figure 1C).

### Differential recruitment of DNA/histone methyltransferases and altered histone modification at the E1 site of *HoxA13* promoter

Because the hypomethylation at the E1 site of *HoxA13* promoter was found in CS12 cells, we next conducted ChIP assays to compare the recruitment of DNA methyltransferases to the E1 site in CS12 and CSN cells. The results showed that the recruitment of DNMT1 and DNMT3b to the E1 site was one-half to one-third lower in CS12 than in CSN cells (Figure 4E), supporting the observed hypomethylation of *HoxA13* promoter even though the expression of DNMT1 and DNMT3b was 1.8-fold higher in CS12 cells than in CSN cells (Supplementary Figure 5).

Next, we examined the recruitment of MLL1 and WDR5 and the methylation of histone H3 at the E1 site in CS12 and CSN cells because the previous study showed that MLL1/WDR5 complex mediates trimethylation of histone H3 lysine 4 (H3K4me3) at the 5′- *HoxA* cluster and activates *HOXA* gene expression. The results showed that MLL1 and WDR5 densely occupied at the E1 site but not at the NS site, which was coincident with increased H3K4me3 and HoxA13 expression in CS12 but not in CSN cells (Figures 2 and 4E).

### The recruitment of lncRNA HOTTIP was involved in the upregulation of HoxA13 in CS12 cells

**Figure 5:**
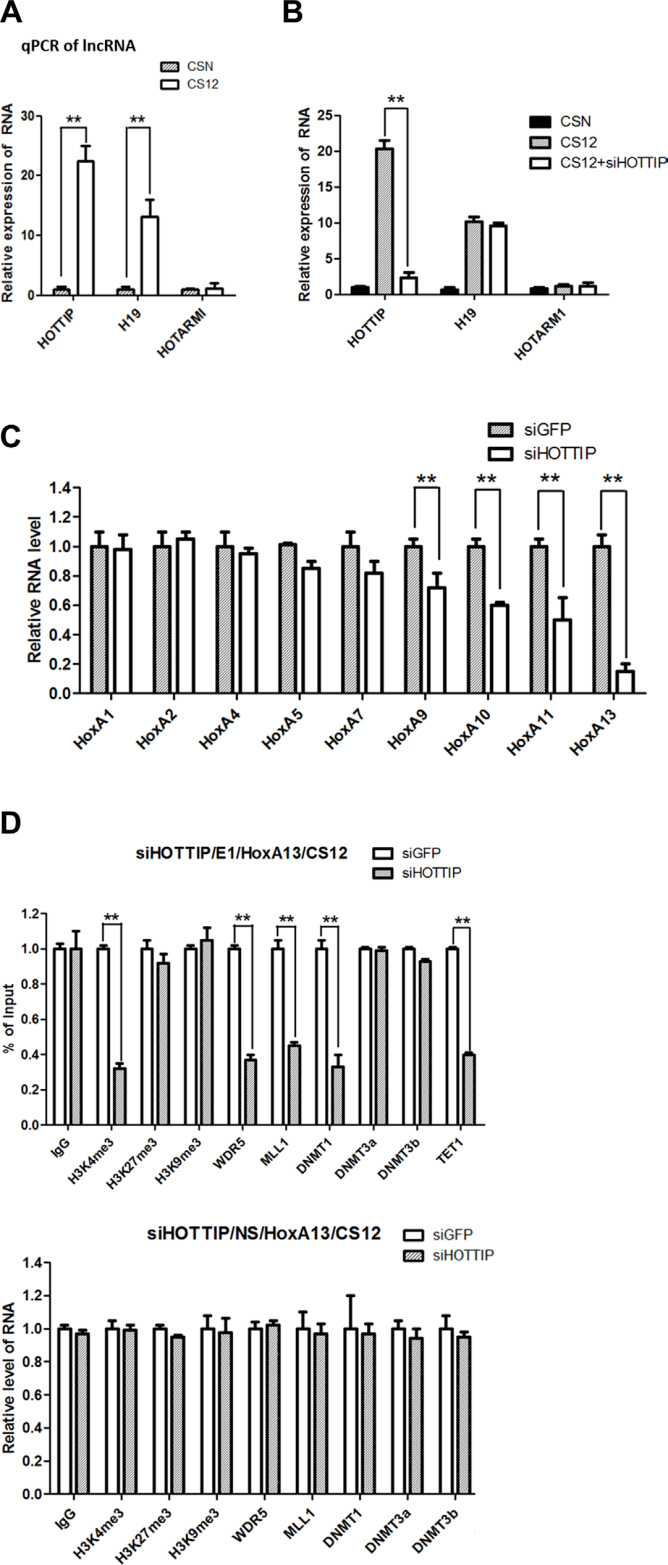
The epigenetic role of *HOTTIP* in the E1 site of *HoxA13* promoter (**A**) Relative expression of long noncoding RNA (lncRNA) in CSN and CS12 cells. The expression of lncRNA in CSN cells was taken as 1.0. (**B**) Effect of siRNA to HOTTIP on the expression of lncRNAs in CSN and CS12 cells. (**C**) Effect of siRNAs to HOTTIP on the expression of the *HOXA* gene family. Each expression of siRNA-GFP treated CS12 cells was taken as 1.0. (**D**) Effect of siRNA-HOTTIP on recruitment of DNA methyltransferases, WDR5, MML1, and methylation of histones at the E1 and NS sites of the *HoxA13* promoter. Each expression of siRNA-GFP treated cells was taken as 1.0. All data are presented as mean ± SEM (two-tailed Student *t* test; **p < 0.01).

The recruitment of the WDR5–MLL complex to the 5′-end *HOXA* cluster is mediated by the lncRNA HOTTIP [19, 20]. The q-PCR results showed that the expression level of HOTTIP was 17.8–25.1-fold higher in CS12 cells than in CSN cells (Figure 5A). However, the expression level of HOTARMI, the lncRNA resides on the 3′-end *HoxA* cluster, was similar between both cells. Interestingly, another lncRNA H19 was also highly expressed in CS12 cells.

To confirm the involvement of HOTTIP in the upregulation of HoxA13 in CS12 cells, siRNA against *HOTTIP* was introduced to reduce the HOTTIP, but not the H19 RNA levels (Figure 5B). Upon *HOTTIP* knockdown, the expression of 5′-end *HOXA* genes including *HoxA13* were reduced in CS12 cells when compared with the effect of control siRNA (Figure 5C). ChIP analyses showed that both the recruitment of WDR5 and MLL1 and the level of H3K4me3 at the E1 site, but not at the NS site, were decreased in CS12 cells after *HOTTI* knockdown (Figure 5D). In addition, the recruitment of DNMT3b, but not DNMT1, was restored by *HOTTIP* knockdown at the E1 site. Thus, *HOTTIP* might affect the recruitment of DNA methyltransferase DNMT3b but not DNMT1. These results demonstrated that *HOTTIP* was involved in the upregulation of HoxA13 in CS12 cells.

### IGFBP-3 was a HoxA13 downstream target and was important for the cancerous features of CS12 cells

**Figure 6:**
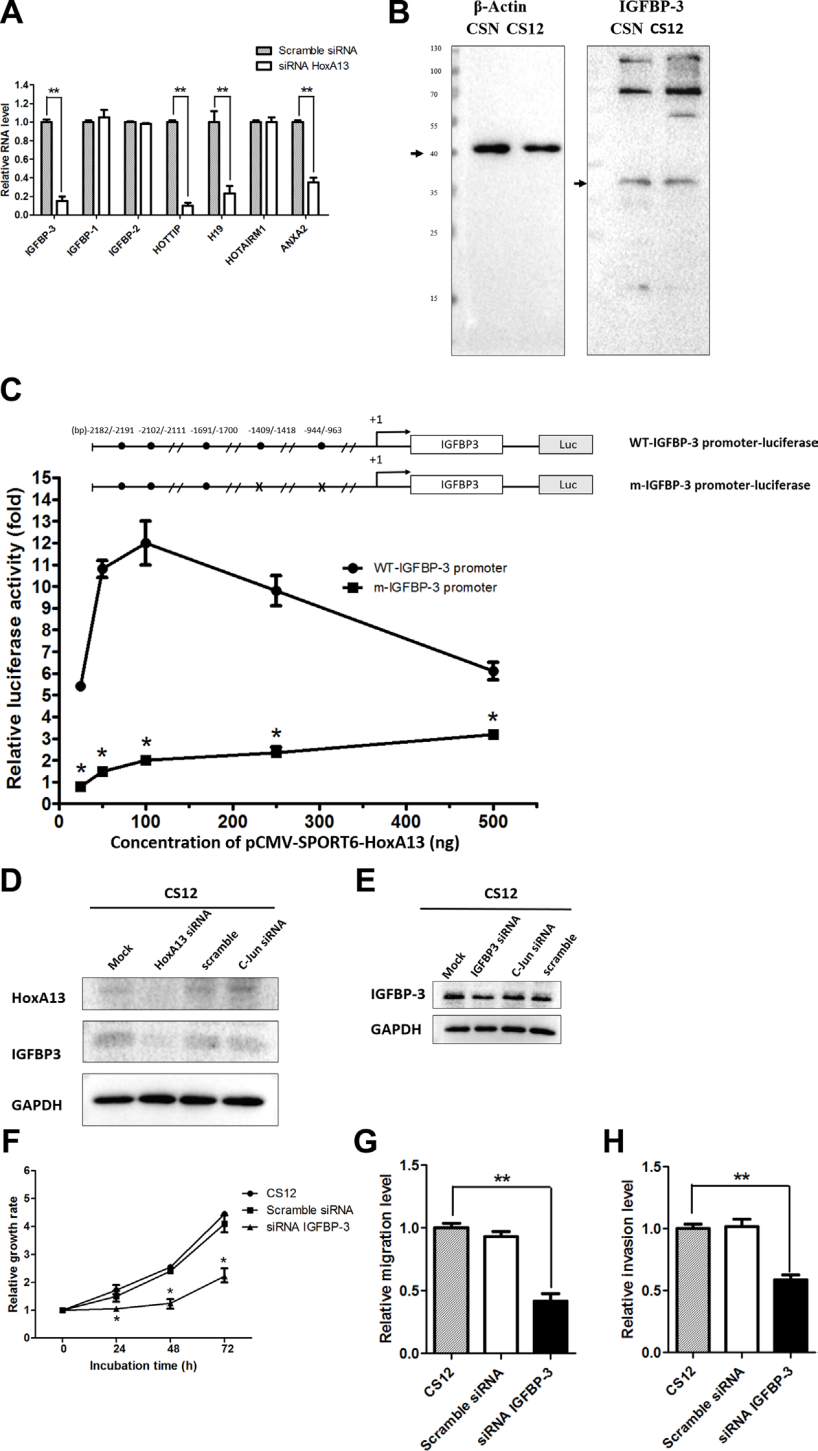
*IGFBP-3* and *HOTTIP* are downstream gene to HoxA13 in CS 12 cells (**A**) Effect of siRNA-HoxA13 on mRNA expression of IGFBP family and lncRNAs was examined by qPCR in CS12 cells. Each expression level of scramble-siRNA treated CS12 cells was considered to be 1.0. (**B**) Expression of IGFBP-3 protein was examined by western blot in CSN and CS12 cells. (**C**) The luciferase constructs of WT-IGFBP-3 promoter and its mutant-IGFBP-3 promoter with various amounts of pcDNA3-HoxA13 (25 ng, 50 ng, 100 ng, 250 ng, and 500 ng) were transfected into CS12 cells. Two days after transfection, cells were harvested and luciferase activity was measured as described in the Materials and Methods. The luciferase activity of m-IGFBP-2 promoter-luciferase in the presence of 25 ng of pcDNA3-HoxA13 was considered to be 1.0. (**D**) Expression of IGFBP-3 in CS12 cells treated with siRNA-HoXA13 or scramble or off target siRNA was assessed as described in Materials and Methods. (**E**) Expression of IGFBP-3 in CS12 cells treated with siRNA-IBGBP-3 or scrambled siRNA was examined by western blot. (**F**) The effect of siRNA-IGFBP-3 on cell growth in CS12 cells was assessed as described in the Materials and Methods. (**G**), (**H**) The effect of siRNA to IGFBP-3 on migration (G) and invasion (H) activities was assessed in described in the Materials and Methods. The value of CS12 was considered to be 1.0. Data were derived from five independent experiments and presented as mean ± SEM (two-tailed Student *t* test; *p < 0.05, **p < 0.01).

To investigate the mechanism underlying HoxA13 downstream genes including those for Annexin A2 (ANXA2) [34, 35] and IGFBP-3. siRNA against *HoxA13* decreased the expression of HOTTIP, H19, IGFBP-3, and ANXA2 in CS12 cells (Figure 6A). The endogenous expression of IGFBP-3 was about two- to threefold higher in CS12 than in CSN cells (Figure 6B). To verify the effect of HoxA13 on IGFBP-3 expression, *IGFBP-3* promoter was cloned to a luciferase reporter, and then cotransfected with a HoxA13 expressing construct or vector control into CSN cells. The results showed that ectopic HoxA13 expression significantly activated *IGFBP-3* promoter activity (Figure 6C). However, this transactivation was impaired by mutation of two putative HOX-binding sites on the *IGFBP-3* promoter. These results confirmed that IGFBP-3 was a HOXA13 target gene. We also examined the effect of HoxA13 on the expression of IGFBP-3 in *HoxA13* siRNA transfected CS12 cells and found that *HoxA13* siRNA reduced the expression of IGFBP-3 by 80 to 85%, but scramble and off-target siRNA did not show the significant reduction (Figure 6D). To search for IGFBP-3 mediated cancer related activity, we constructed *IGFBP-3* siRNA. The expression of IGFBP-3 in *IGFBP-3* siRNA-transfected CS12 cells was significantly reduced, but the scrambled and off-target siRNA did not change the expression of IGFBP-3 (Figure 6E). Importantly, siRNA against *IGFBP-3* reduced cell growth of CS12 cells significantly, but scrambled siRNA did not reduce cell growth (Figure 6F). Knockdown of *IGFBP-3* also reduced migration (Figure 6G) and invasion (Figure 6H) activities of CS12 cells. These results suggest that IGFBP-3 is critical for the cancerous features of CS12 cells.

### Increased expression of HoxA13 and IGFBP-3 in human gastric cancer

**Figure 7:**
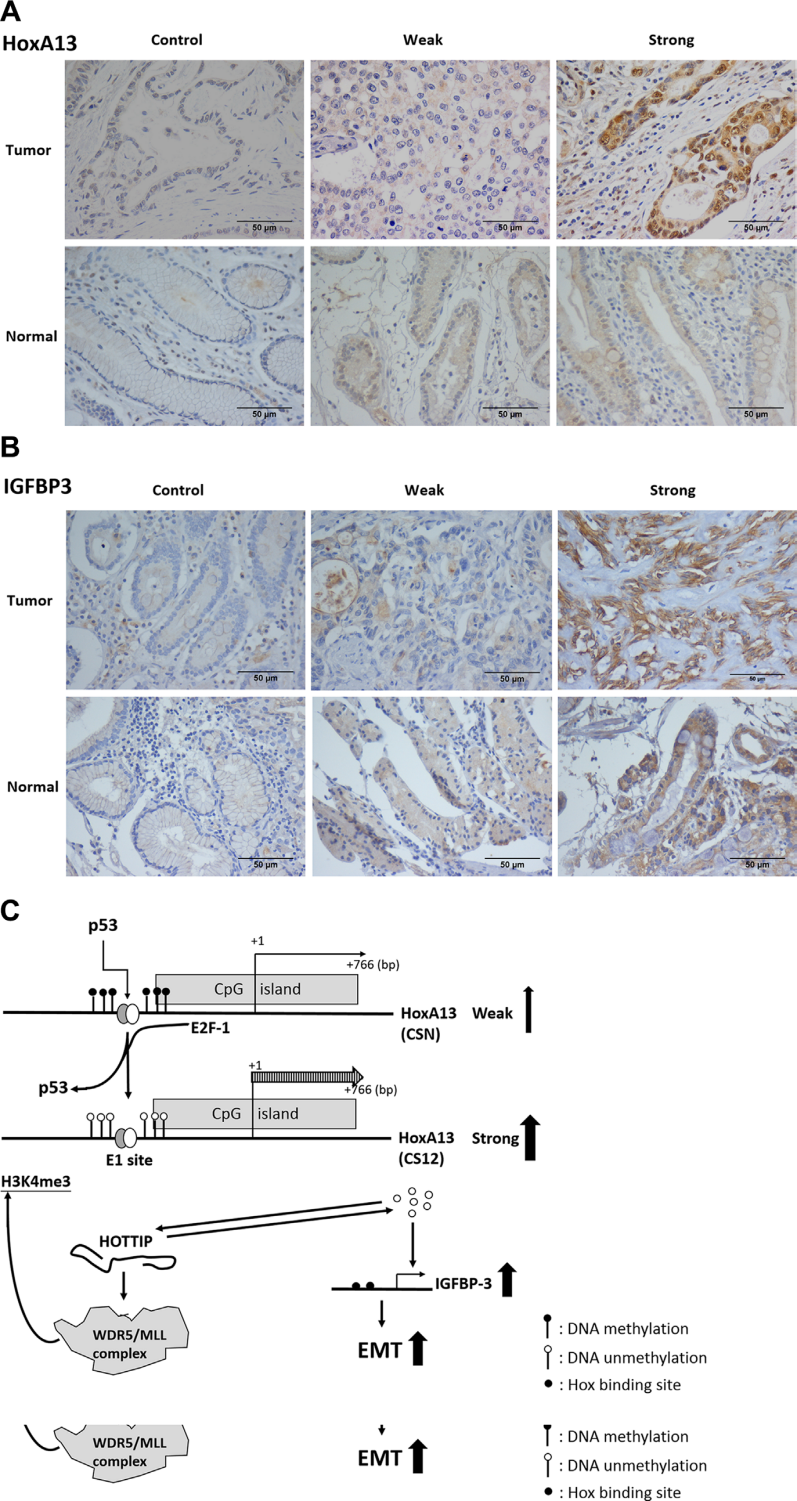
Expression of HoxA13 and IGFBP-3 in specimens of gastric cancer patients (**A**, **B**) Representative immunohistochemical staining shows strong (score > 4), weak (score < 2) and non (isotype control) expression of HoxA13 (A) and IGFBP-3 (B). (**C**) Schematic representation of the HoxA13–HOTTIP–IGFBP-3 axis during the development of gastric cancer. EMT: epithelial mesenchymal transition.

The expression of HoxA13 was examined by immunohistochemistry (IHC) in gastric cancer obtained from 57 patients. HoxA13 was detected in 73.7% (42/57) of these samples (Figure 7A and 7B). The expression of IGFBP-3 was stained for 28 specimens and the positive rate was 50.0% (14/28). By scoring expression levels (0 to 7) by positivity and intensity of IHC staining, HoxA13 and IGFBP-3 were highly expressed (score ≥ 4) in 63.2% (36/57) and 28.6% (8/28), respectively, of these gastric cancer specimens (Supplementary Table 1). We also analyzed HoxA13 and IGFBP-3 expression using the gastric cancer data of The Cancer Genome Atlas and found that both genes were overexpressed (Supplementary Figure 6).

## DISCUSSION

We found DNA hypomethylation at the p53–E2F-1 responsive element (E1 site) of the *HoxA13* promoter in CS12 cells when compared with CSN cells, together with increased recruitment of E2F, but exclusion of p53 binding at the E1 site, which were consistent with increased HoxA13 expression in CS12 cells. Forced expression of E2F-1 activated, but p53 inhibited HoxA13 promoter in CS12 and CSN cells (Figure 4D, Supplementary Figure 3). The identification of differential DNA methylation at the E1 site of the *HoxA13* promoter in CSN and CS12 cells is a new finding, which provides a mechanism to explain the upregulation of *HoxA13* expression during the development of gastric cancer (Figure 2). Moreover, expression of *HoxA13* was observed in gastric cancer specimen (Figure 7A, Supplementary Figure 6, and Supplementary Table 1). One previous study reported that increased HoxA13 expression was a poor prognostic factor in gastric cancer [17], this study further demonstrated that HoxA13 enhanced the migration and invasion ability of gastric cancer cells.

The hypomethylation at the E1 site of the *HoxA13* promoter in CS12 cells was concurrent with decreased recruitments of the DNA methyltransferases DNMT1 and DNMT3b (Figure 4E). In addition, increased binding of WDR5 and MLL complex together with elevated levels of H3K4me3 were observed at the E1 site, which were dependent on increased HOTTIP expression in CS12 cells (Figure 5D). These result showed that HOTTIP activated HoxA13 expression through epigenetic mechanism including DNA methylation and histone modification. Interestingly, knockdown of HoxA13 led to a decrease of HOTTIP expression (Figure 6), demonstrating a positive feedback control of HoxA13 and HOTTIP expression.

More than 230 sequence-specific and specially expressed lncRNAs have been reported to be associated with the *HOX* gene family [39]. In liver, pancreas, and tongue squamous cell cancers, HOTTIP is positively associated with HoxA13 expression [20, 22, 23].

Up-regulation of HOTTIP is a negative prognostic factor for hepatocellular carcinoma patients [20]. Overexpression of HOTTIP in human pancreatic cancers increases cell proliferation, invasion, and EMT activity [23]. These studies support the oncogenic role of HOTTIP, which induces expression of another oncogene HoxA13 in gastric cancer.

We identified both IGFBP-3 and HOTTIP are the target genes of HoxA13 in gastric cancer. HoxA13 transactivated IGFBP-3 gene expression via Hox-binding elements in the *GFBP-3* promoter (Figure 6C). Despite extensive investigation showing the involvement of IGFBP-3 in cancers, it is not a currently used cancer biomarker because it is debatable whether IGFBP-3 is up- or down-regulated in cancers. In gastric cancer, IGFBP-3 has been reported to be a suppressor of migration, invasion, and the EMT through suppression of invasive factors including MMP14 and urokinase-type plasminogen activator [40]. We here found contradictory results in CS12 cells, where both the expression of IGFBP-3 and cell migration were increased significantly. Besides, knockdown of IGFBP-3 inhibited cell proliferation, colony formation, migration, and invasion (Figure 6E–6G). IGFBP-3 may potentiate gastric cancer cell division and invasion that contradicts previous findings of its role as a tumor suppressor [26]. There may be multiple factors that can influence IGFBP-3 expression, and its expression may have both positive and negative effects on tumor development as reported previously [24, 25, 41].

OCT4 was expressed in CSN and CS12 cells, indicating that both cell lines may have the stemness characteristics [36]. However, only CS12 expressed NANOG and LGR5 (Supplementary Figures 1A, 1B, and 2C) and expressed higher levels of SOX2, NANOG, REX1, and hTERT than CSN cells (Supplementary Figure 2D). Only CS12 cells showed differentiation of two germ layers and exhibited strong tumor formation of xenografts in SCID mice (Supplementary Figure 2E). Thus, we conclude that CS12 cells bear the cancer stem cell-like characteristics including characteristic patterns of cell proliferation, cell cycle progression, invasion and migration, and drug resistance. However, CS12 cells are not stem cells because the staining of alkaline phosphatase is negative.

In conclusion, the previously established gastric cancer CS12 cell line showed the characteristics of stemness gene expression but was not fully pluripotent because only two germ layers were differentiated. We also identified the IGFBP-3 as the target of HoxA13 and a positive regulator of gastric cancers. Both HoxA13 and IGFBP-3 were overexpressed significantly in human gastric cancer specimens of Taiwan (Supplementary Table 1) and The Cancer Genome Atlas (Supplementary Figure 6). Thus, the HoxA13–HOTTIP–IGFBP-3 axis might be an oncogenic pathway in the gastric cancer and a potential new oncotarget for gastric cancer therapy.

## MATERIALS AND METHODS

### Cell lines, reagents, and animals

Human gastric normal cells CSN and cancer cells CS12 cells were cultured as described elsewhere [30] with a slight modification to include Keratinocyte-SFM (Gibco-Invitrogen Co., Carlsbad, CA, USA). 293T cells were obtained from the RIKEN Cell Bank (Tsukuba, Ibaraki, Japan) and were cultured in Dulbecco's modified Eagle's minimal essential medium (DMEM) (Gibco) supplemented with 10% charcoal-stripped FBS (Gibco) with or without 1% penicillin and streptomycin (Gibco). The animal welfare guidelines for the care and use of laboratory animals were approved by the Animal Care Committee of the RIKEN BioResource Center in Japan, the National Laboratory of Animal Center and the Kaohsiung Medical University in Taiwan.

### Patient samples

This study enrolled patients with gastric cancers from the Kaohsiung Medical University Hospital (KMUH) from June 2010 to August 2013. The study of human subjects was approved by the Institutional Review Board of the KMUH (KMUHIRB-960343, Kaohsiung, Taiwan). All patients gave their informed consent, and the ethics and scientific committees of the participating institutions approved the study. Tumor types were determined according to the World Health Organization classification. At the time of surgery, all tissue samples were immediately flash-frozen in liquid nitrogen and stored at −80°C until use. Patient samples were stained with antibodies against HoxA13 and IGFBP-3 as described elsewhere [30, 31].

### Plasmids, small interference RNA (siRNA) and short hairpin RNA (shRNA) lentivirus

The expression plasmids of human HoxA13 cDNA and HoxA13 promoter were obtained from the RIKEN DNA Bank (IRAK168L10; Tsukuba, Ibaraki, Japan) and Active motif (NM000522.4; Carlsbad, CA, USA), and inserted into the pcDNA3 (Invitrogen-Thermo Fisher Scientific, Waltham, MA, USA) and pGL4 luciferase vectors (Promega Corp., Madison, WI, USA), respectively, to generate pcDNA–Flag–HoxA13 and HoxA13 promoter-luciferase. The mutant of E1 of HoxA13 promoter was generated by polymerase chain reaction (PCR) using the primer of 5′-ATGAACAACCACCCTAACACAAC-3′. Human IGFBP-3 promoter [−2,282 nucleotide (nt) to +56 nt]-luciferase and its series of mutants were gifts by Dr. T. Hanafusa (Okayama, Univresity) [27]. All constructs were confirmed by DNA sequencing. The HoxA13 shRNA lentivirus, the GFP shRNA or scrambled shRNA lentivirus were generated in 293T cells that had been cotransfected with pCAG-HIVgp pCMV-VSV-G-RSV-Rev, and TRCN0000004881 (Academia Sinica, Taipei, Taiwan) or PLKO.1-GFP (#30323; Addgene, Cambridge, MA, USA) or scrambled control shRNA (Sigma-Aldrich, St. Louis, MO, USA). Virus supernatants were collected 72 h after transfection, and particles were purified as described [42]. The CS12 cells (1 × 10^6^) were infected with shRNA HoxA13 lentiviruses or scrambled shRNA at a multiplicity of infection of 4. After cultivation for 3 days, cells were injected into SCID mice (5 × 10^6^ cells/spot). For siRNA-mediated gene knockdown, cells were transfected with negative control siRNA (Thermo Fisher Scientific, D-001810-10-05) or the following specific siRNA-like IGFBP-3-targeting siRNA (Ambion-Thermo Fischer, s7227, s7228, s7229), HoxA13-targeting siRNA (Ambion-Thermo Fisher; s106130, s6785, 6886, 6787), HOTTIP-targeting siRNA (Sigma-Aldrich, LQ-011052-00-0002) or c-JUN-targeting siRNA (Ambion-Thermo Fischer: s7658) using Lipofectamine RNA/MAX reagents (Thermo Fisher) [43–45]. All sequences were run on BLAST, to exclude sequences that would suppress undesired genes and to ensure specificity. The cells were harvested after 48 h of incubation, and the effects of the compound alone on gene expression were assessed.

### Cell proliferation, colony assay and cell cycle analyses

The living cells were counted using the trypan blue dye-exclusion method, and were analyzed by flow cytometry to identify the sub-G population of cells [43]. MTT assay was assayed as followed to the manufacturers' instructions as described elsewhere [44, 45]. A colony assay was performed as described elsewhere [43]. Briefly, cells were plated in duplicate at 5 × 10^2^ or 5 × 10^3^ cells per gelatin-coated dish. Two weeks later, colonies with a diameter > 2 mm were counted after staining with Giemsa staining solution (Wako Chemical Co., Tokyo, Japan). For analysis of the cell cycle [46], serum-starved cells were cultured in DMEM containing 15% FBS and collected at the indicated times. Harvested cells were stained with propidium iodide (PI; 1 μg/mL), and subjected to a fluorescence-activated analysis of DNA content in a flow cytometer (EPICS XL-MCL; Beckman Coulter, Miami, FL, USA).

### Migration, invasion, and chmoresistance assays

Cells (1 × 10^4^ cells) cultured in DMEM without FBS were seeded in the upper Transwell plate coated with or without matrix gel (Corning, Inc., NY, USA; 1 mg/mL). The lower plate contained DMEM plus 10% FBS. Three days later, the cells on the lower plate of the Transwell were fixed with 4% formaldehyde, stained with 1% crystal violet, and the cells were counted under a microscope. Regarding chemoresistance assay, cells were seeded in 96-well plates and incubated for 24 h, to allow cell attachment. DMEM containing a serial dilution of 5-fuluorouracil (20 μg/mL) was added, and the cells were incubated for an additional 48 h in 5% CO. Cell viability was examined using the MTT assay.

### Teratoma formation assay, alkaline phosphatase and immunohistochemistry

Induced pluripotent stem cells (iPS-like cells) (200 cells; one colony/spot) were injected subcutaneously into the dorsal flank of severe combined immunodeficiency (SCID) mice, as described elsewhere [47]. The teratomas that formed after the injection were fixed in 4% paraformaldehyde overnight and embedded in paraffin. Sections were stained with hematoxylin and eosin. Measurement of alkaline phosphatase activity and immunocytochemistry were performed as described elsewhere [47]. The antibodies used in this work were listed in Supplementary Table 2.

### Immunoprecipitation and western blotting

Immunoprecipitation and western blotting were performed as described elsewhere [48, 49].

### Reverse transcriptase PCR (RT-PCR) and quantitative polymerase chain reaction (qPCR)

Total RNA was extracted from cells using the TRIzol reagent (Thermo Fisher scientific). RNA was reverse transcribed to cDNA using a reverse transcription kit (Promega). PCR was performed using the GoTaq^®^ green master mix (Promega). qPCR was performed using the Quantifast SYBR green PCR kit (Qiagen, Gaithersburg, MD, USA) as described elsewhere [43–46]. Amplification curves and gene expression were normalized to those of β-actin or GAPDH which was used as an internal control. The primers used for qPCR are listed in the Supplementary Tables 3–6.

### Transient transfection and luciferase assay

Transient transfection and luciferase assay were performed as described [43–46]. Cells were plated into each well of a 12-well plate and cultured for 24 h. The cells were then cotransfected with the indicated amount of constructs carrying the IGFBP3-promoter, HoxA13-promoter-luciferase reporters and with or without increasing dose of HoxA13, using Lipofectamine 2000 (Invitrogen). The total amount of transfected DNA was kept constant at 1 μg/well by the addition of pBluescript. After 48 h or the indicated period of incubation, the cells were harvested and the activities of luciferase were measured in an illuminometer (Berthold Technologies GmbH and Co. KG, Bad Wildbad, Germany) using the Dual-Luciferase Reporter Assay System (Promega). Luciferase activity values were normalized to transfection efficiency.

### Quantitative DNA methylation analysis by mass ARRAY epityping

High molecular weight DNA was isolated from CSN and CS12 cells using the PureGene kit from Qiagen (Hilden, Germany). Bisulfite conversion was performed by using the EZ DNA Methylation-Gold kit (Zymo Research Co., Irvine, CA, USA, Cat. no. D5005) [49]. The promoter region of the HoxA13 gene was PCR-amplified from bisulfite-treated human genomic DNA using primers that incorporated the T7 promoter sequence. The DNA methylation analysis was performed on Mass ARRAY (Sequenon) through MALDI-TOF mass spectrometer for data acquisition [50]. Comparative sequence analysis was done by using EpiTYPER software for the percentage of specific site methylation [47]. MassAray primers were designated to cover the promoter regions of the indicated genes.

### Chromatin immunoprecipitation assay (ChIP)

The ChIP assay was performed as described elsewhere [46]. The immunoprecipitated protein–DNA complexes were washed twice with binding buffer (10 mM HEPES, pH 7.9, 10 mM Tris-HCl, pH 7.9, 12.5% glycerol, 0.25% NP-40, 0.5% Triton X-100, 0.24 M NaCl, 0.75 mM MgCl_2_, 1.1 mM EDTA, and protease inhibitor mixture) and then washed twice with Tris-EDTA buffer (10 mM Tris-HCl, pH 7.9, and 1 mM EDTA). The protein–DNA complexes were disrupted with proteinase K (Sigma-Aldrich) DNA was extracted with phenol and chloroform, precipitated in ethanol, and analyzed by real-time PCR using the Power SYBR Green Master Mix (Invitrogen). The PCR conditions consisted of 1 cycle of 2 min at 50°C and 1 cycle of 10 min at 95°C followed by 40 cycles of 95°C for 15 sec, and 55–60°C for 60 sec. The primers used in these experiments are shown in the Supplementary Table 7.

### Statistical analysis

The data are presented as the mean ± SEM from triplicate experiments and additional replicates as indicated. Significance was assessed using two-way ANOVA (*P* < 0.0001) followed by two-tailed student's *t*- tests. Survival analysis was performed using the Kaplan–Meier method, and the curves were compared using the log-rank test. A *P* value < 0.05 was considered statistically significance.

## SUPPLEMENTARY MATERIALS FIGURES AND TABLES


