# Supplementary material for: Oncogenic function of the homeobox A13-long noncoding RNA HOTTIP-insulin growth factor-binding protein 3 axis in human gastric cancer
Source: Oncotarget. 2016 Apr 29;7(24):36049–64. doi: 10.18632/oncotarget.9102 (PMC5094982; doi:10.18632/oncotarget.9102)
Supplement: Supplementary file 1 [file oncotarget-07-36049-s001.pdf]

Oncogenic function of the homeobox A13-long noncoding RNA HOTTIP-insulin growth factor-binding protein 3 axis in human gastric cancer

Supplementary Materials

MATERIALS AND METHODS

Plasmids, small interference RNA (siRNA) and short hairpin RNA (shRNA) lentivirus

The expression plasmids of human HoxA13 cDNA and HoxA13 promoter were obtained from the RIKEN DNA Bank (IRAK168L10; Tsukuba, Ibaraki, Japan) and Active motif (NM000522.4; Carlsbad, CA, USA), and inserted into the pcDNA3 (Invitrogen-Thermo Fisher Scientific, Waltham, MA, USA) and pGL4 luciferase vectors (Promega Corp., Madison, WI, USA), respectively, to generate pcDNA–Flag–HoxA13 and HoxA13 promoter-luciferase. The mutant of E1 of HoxA13 promoter was generated by Polymerase chain reaction (PCR) using the primer of 5′-ATGAACAACCACCCTAACACAAC-3′. Human IGFBP-3 promoter [–2,282 nucleotide (nt) to +56 nt]-luciferase and its series of mutants were gifts by Dr. T. Hanafusa (Okayama, Univresity) [1]. All constructs were confirmed by DNA sequencing. The HoxA13 shRNA lentivirus, the GFP shRNA or scrambled shRNA lentivirus were generated in 293T cells that had been cotransfected with pCAG-HIVgp, pCMV-VSV-G-RSV-Rev, and TRCN0000004881 (Academia Sinica, Taipei, Taiwan) or PLKO.1-GFP (#30323; Addgene, Cambridge, MA, USA) or scrambled control shRNA (Sigma-Aldrich, St. Louis, MO, USA). Virus supernatants were collected 72 h after transfection, and particles were purified as described [2]. The CS12 cells (1×10^6) were infected with shRNA HoxA13 lentiviruses or scrambled shRNA at a multiplicity of infection of 4. After cultivation for 3 days, cells were injected into SCID mice (5×10^6 cells/spot). For siRNA-mediated gene knockdown, cells were transfected with negative control siRNA (Thermo Fisher Scientific, D-001810-10-05) or the following specific siRNA-like IGFBP-3-targeting siRNA (Ambion-Thermo Fischer, s7227, s7228, s7229), HoxA13-targeting siRNA (Ambion-Thermo Fisher; s106130, s6785, 6886, 6787) or HOTTIP-targeting siRNA (Sigma-Aldrich, LQ-011052-00-0002) or c-JUN-targeting siRNA (Ambion-Thermo Fischer: s7658) using Lipofectamine RNA/MAX reagents (Thermo Fisher) [3–5]. All sequences were run on BLAST, to exclude sequences that would suppress undesired genes and to ensure specificity. The cells were harvested after 48 h of incubation, and the effects of the compound alone on gene expression were assessed.

Cell proliferation, colony assay and cell cycle analyses

The living cells were counted using the trypan blue dye-exclusion method, and were analyzed by flow cytometry to identify the sub-G_1 population of cells [3]. MTT assay was assayed as followed to the manufacturers' instructions as described elsewhere [4, 5]. A colony assay was performed as described elsewhere [3]. Briefly, cells were plated in duplicate at 5×10^2 or 5×10^3 cells per gelatin-coated dish. Two weeks later, colonies with a diameter > 2 mm were counted after staining with Giemsa staining solution (Wako Chemical Co., Tokyo, Japan). For analysis of the cell cycle [6], serum-starved cells were further cultured further in DMEM containing 15% FBS and collected at the indicated times. Harvested cells were stained with propidium iodide (PI; 1 µg/mL), and subjected to a fluorescence-activated analysis of DNA content in a flow cytometer (EPICS XL-MCL; Beckman Coulter, Miami, FL, USA).

Migration, invasion, and chmoresistance assays

Cells (1×10^4 cells) cultured in DMEM without FBS were seeded in the upper Transwell plate coated with or without matrix gel (Corning, Inc., NY, USA; 1 mg/mL). The lower plate contained DMEM plus 10% FBS. Three days later, the cells on the lower plate of the Transwell were fixed with 4% formaldehyde, stained with 1% crystal violet, and subsequently, and the cells were counted under a microscope. Regarding chemoresistance assay, cells were seeded in 96-well plates and incubated for 24 h, to allow cell attachment. DMEM containing a serial dilution of 5-fuluorouracil (20 µg/mL) was added, and the cells were incubated for an additional 48 h in 5% CO_2. Cell viability was examined using the MTT assay.

Teratoma formation assay, alkaline phosphatase and immunohistochemistry

Induced pluripotent stem cells (iPS-like cells) (200 cells; one colony/spot) were injected subcutaneously into the dorsal flank of severe combined immunodeficiency (SCID) mice, as described elsewhere [7]. The teratomas that formed after the injection were fixed in 4% paraformaldehyde overnight and embedded in paraffin. Sections were stained with hematoxylin and

eosin. Measurement of alkaline phosphatase activity measurement and immunocytochemistry were performed as described elsewhere [7].

Immunoprecipitation and western blotting

Immunoprecipitation and western blotting were performed as described elsewhere [3–7].

Reverse transcriptase PCR (RT-PCR) and quantitative polymerase chain reaction (qPCR)

Total RNA was extracted from cells using the TRIzol reagent (Thermo Fisher scientific). RNA was reverse transcribed to cDNA using a reverse transcription kit (Promega). PCR was performed using the GoTaq® green master mix (Promega). qPCR was performed using the Quantifast SYBR green PCR kit (Qiagen, Gaithersburg, MD, USA) as described elsewhere [3–7]. Amplification curves and gene expression were normalized to those of β-actin or GAPDH which was used as an internal control. The primers used for qPCR are listed in the Supplementary Tables 3–6.

Transient transfection and luciferase assay

Transient transfection and luciferase assay were performed as described [3–7]. Cells were plated into each well of a 12-well plate and cultured for 24 h. The cells were then cotransfected with the indicated amount of constructs carrying the IGFBP3-promoter, HoxA13-promoter-luciferase reporters and with or without increasing dose of HoxA13, using Lipofectamine 2000 (Invitrogen). The total amount of transfected DNA was kept constant at 1 μg/well by the addition of pBluescript. After 48 h or the indicated period of incubation, the cells were harvested and the activities of luciferase were measured in an illuminometer (Berthold Technologies GmbH and Co. KG, Bad Wildbad, Germany) using the Dual-Luciferase Reporter Assay System (Promega). Luciferase activity values were normalized to transfection efficiency.

Statistical analysis

The data are presented as the mean ± SEM from triplicate experiments and additional replicates as indicated. Significance was assessed using two-way ANOVA ($P < 0.0001$) followed by two-tailed student's *t*- tests. Survival analysis was performed using the Kaplan–Meier method, and the curves were compared using the log-rank test. A P value < 0.05 was considered statistically significance.

REFERENCES

1. Hanafusa T, Shinji T, Shiraha H, Nouso K, Iwasaki Y, Yumoto E, Ono T, Koide N. Functional promoter upstream p53 regulatory sequence of IGFBP3 that is silenced by tumor specific methylation. BMC cancer. 2005; 5:9.
2. Miyoshi H, Takahashi M, Gage FH, Verma IM. Stable and efficient gene transfer into the retina using an HIV-based lentiviral vector. Proc Natl Acad Sci U S A. . 1997; 94:10319–10323.
3. Pan J, Nakade K, Huang YC, Zhu ZW, Masuzaki S, Hasegawa H, Murata T, Yoshiki A, Yamaguchi N, Lee CH, Yang WC, Tsai EM, Obata Y, et al. Suppression of cell-cycle progression by Jun dimerization protein-2 (JDP2) involves downregulation of cyclin-A2. Oncogene. 2010; 29:6245–6256.
4. Kawasaki H, Song J, Eckner R, Ugai H, Chiu R, Taira K, Shi Y, Jones N, Yokoyama KK. p300 and ATF-2 are components of the DRF complex, which regulates retinoic acid- and E1A-mediated transcription of the c-jun gene in F9 cells. Genes & Dev. 1998; 12:233–245.
5. Kawasaki H, Eckner R, Yao TP, Taira K, Chiu R, Livingston DM, Yokoyama KK. Distinct roles of the co-activators p300 and CBP in retinoic-acid-induced F9-cell differentiation. Nature. 1998; 393:284–289.
6. Tanigawa S, Lee CH, Lin CS, Ku CC, Hasegawa H, Qin S, Kawahara A, Korenori Y, Miyamori K, Noguchi M, Lee LH, Lin YC, Steve Lin CL, et al. Jun dimerization protein 2 is a critical component of the Nrf2/MafK complex regulating the response to ROS homeostasis. Cell Death Dis. 2013; 4:e921.
7. Wang SW, Wang SS, Wu DC, Lin YC, Ku CC, Wu CC, Chai CY, Lee JN, Tsai EM, Lin CL, Yang RC, Ko YC, Yu HS, et al. Androgen receptor-mediated apoptosis in bovine testicular induced pluripotent stem cells in response to phthalate esters. Cell Death & Dis. 2013; 4:e907.

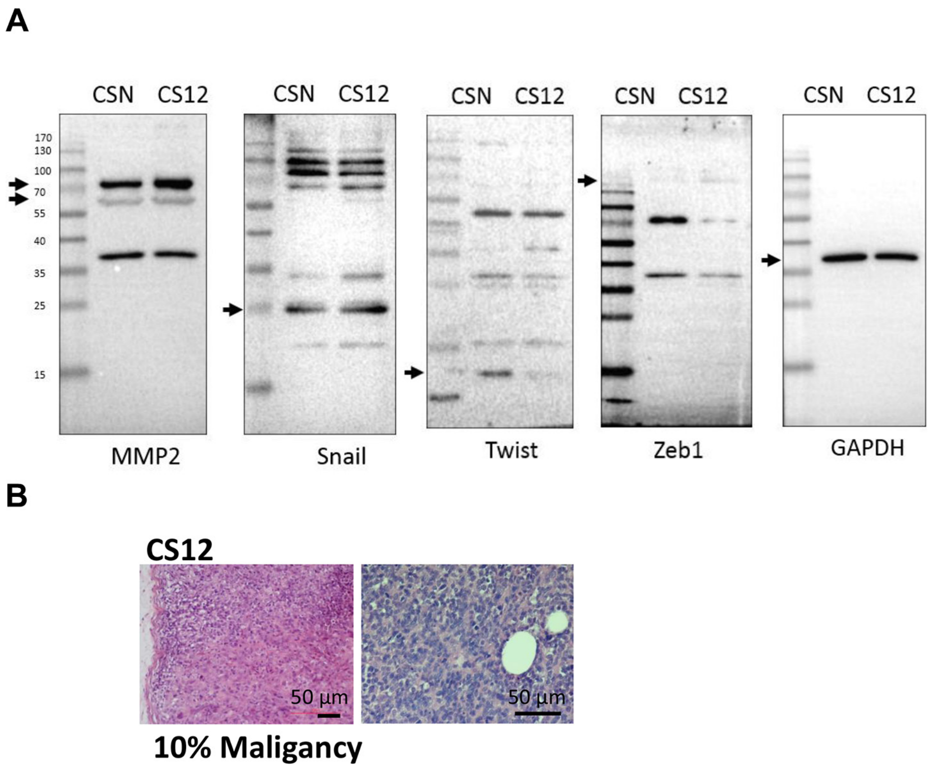

Supplementary Figure S1: Comparative expression of EMT related marker proteins between CSN and CS12 cells and HE staining of tumor in SCID mice derived from CS12. (**A**) Western blots of EMT related proteins in CSN and CS12 cells. Arrowhead is the position of the indicated protein. (**B**) Tumor morphology of transplants in SCID mice subcutaneously injected with CS12 cells. 10% of CS12 sample shows the malignancy. The bar indicated 50 µm.

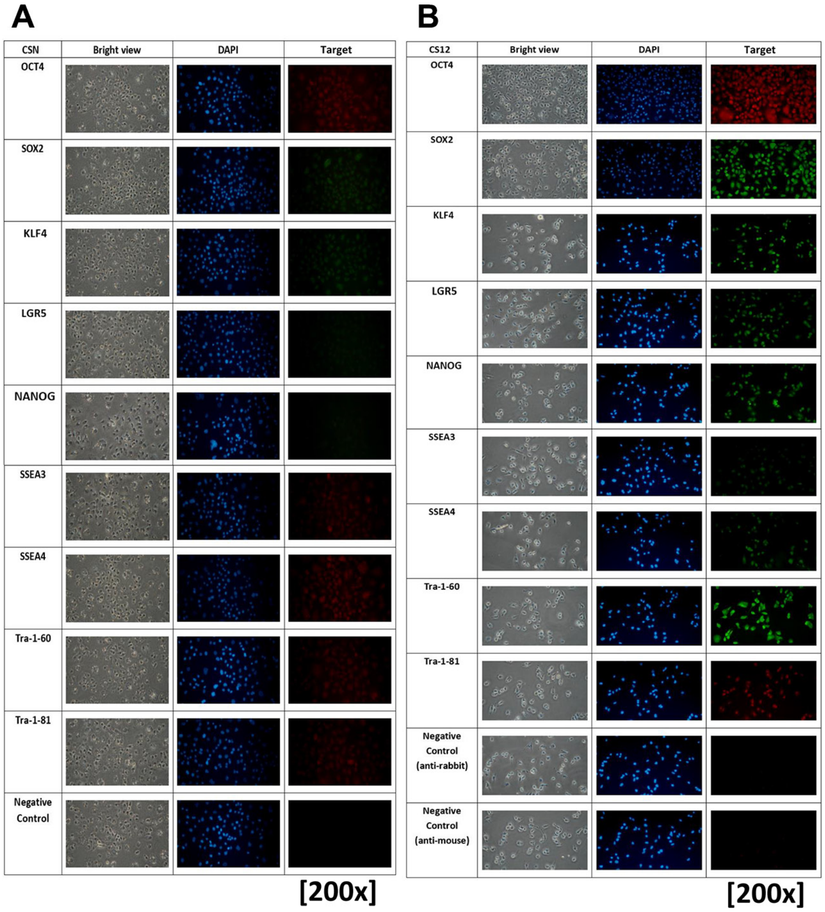

C

	CSN	CS12
OCT4	+	+
SOX2	+	+
KLF4	+	+
LGR5	–	+
NANOG	–	+
SSEA3	+	+
SSEA4	+	+
TRa-1-60	+	+
TRa-1-81	+	+

D

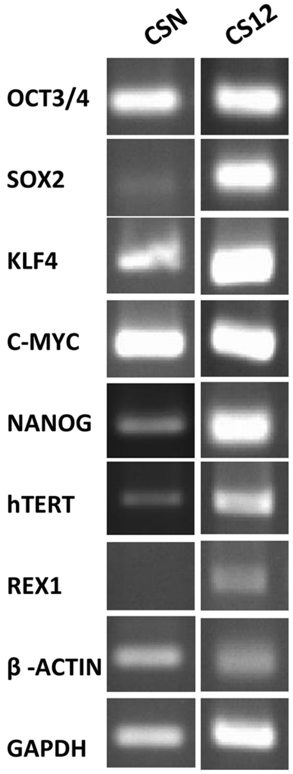

E

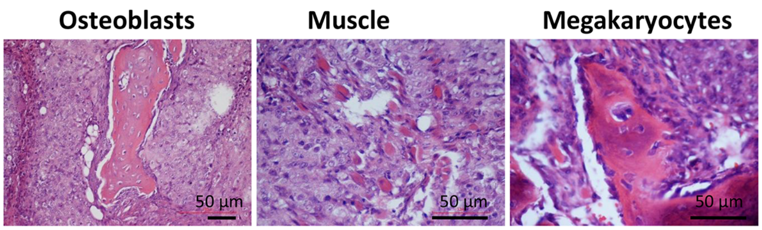

Supplementary Figure S2: Comparative characterization of CSN and CS12 cell lines. (**A**, **B**) Morphology and immunostaining of NANOG and LGR5 in CSN cells (A) and CS12 cells (B). Negative control is the isotype of the used indicated antibodies. DNA was stained with 4′, 6-diamino-2-phenylindole (DAPI) and red fluorescence protein (RFP)/green fluorescence protein (GFP) indicate the secondary RFP and GFP antibodies, respectively. The bars indicated 50 µm. (**C**) Summary of expression of stemness markers in CSN and CSA12 cells. LGR5 and NANOG were not expressed in CSN normal cells. (**D**) Semiquantitative RT-PCR study of stemness genes in CNS and CS12 cells. SOX2, NANOG, hTERT, and REX 1 genes are expressed at significantly higher levels in CS12 cells compared with CSN cells. (**E**) Pluripotency of human CS12 cells. Teratoma formation 6–8 weeks after the transplantation of CS12 cells into SCID mice was investigated. Teratoma were sectioned and stained with hematoxylin and eosin. Osteoblasts, muscle cells and megakaryocytes were detected. Histology of tumor slices derived from CS12 cells showed about 10% of cells exhibited malignancy. The observed higher nucleus/cytoplasm ratio is a typical feature of the cancer phenotype. The bars indicate as 50 µm.

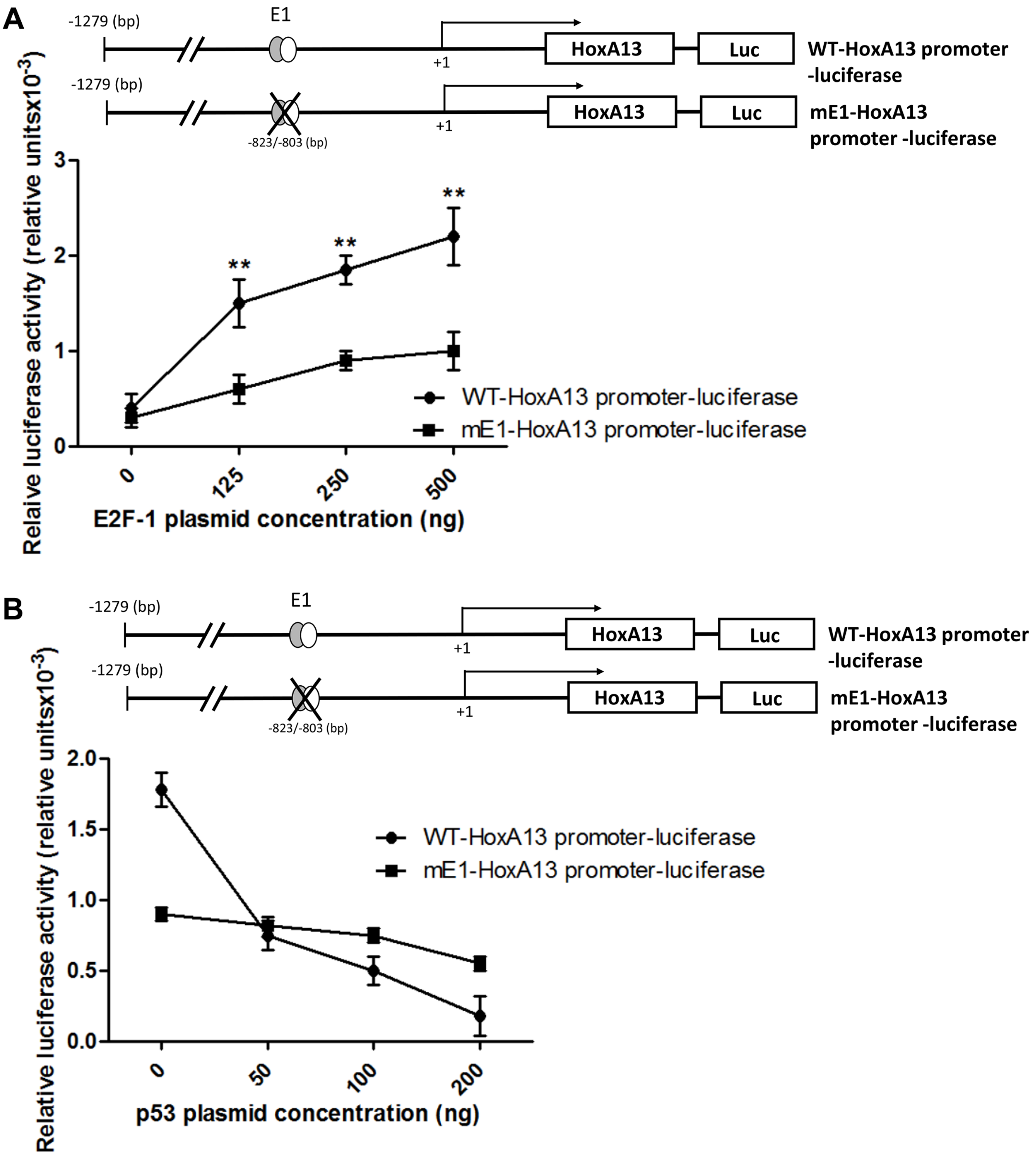

Supplementary Figure S3: Effect of E2F-1 and p53 on HoxA13 promoter activity in CSN cells. Schematic representation of WT HoxA13 promoter and mE1-HoxA13 promoter constructs. E1 elements were mutated as described in Materials and Methods. Effect of E2F-1 expression vector was examined as transaction activity. WT-HoxA13 promoter-luciferase and mE1-HoxA13 promoter-luciferases and E2F-1 expression vector pCMV-SPORT6-E2F-1(0, 125, 250 and 500 ng) were transfected into CSN cells, and the luciferase activity was measured. The values are expressed as means ± S.D. and *t* tests were used to compare these values ($n = 5$, $^{**}P < 0.01$). (**B**) Effect of p53 expression vector was examined. WT-HoxA13 promoter-luciferase and mE1-HoxA13 promoter-luciferases and p53 expression vector pCAG-p53 WT (0, 50, 100 and 200 ng) were transfected into CSN cells, and the luciferase activity was measured. The values are expressed as means ± S.D. and *t* tests were used to compare these values ($n = 3$, $^{**}P < 0.01$).

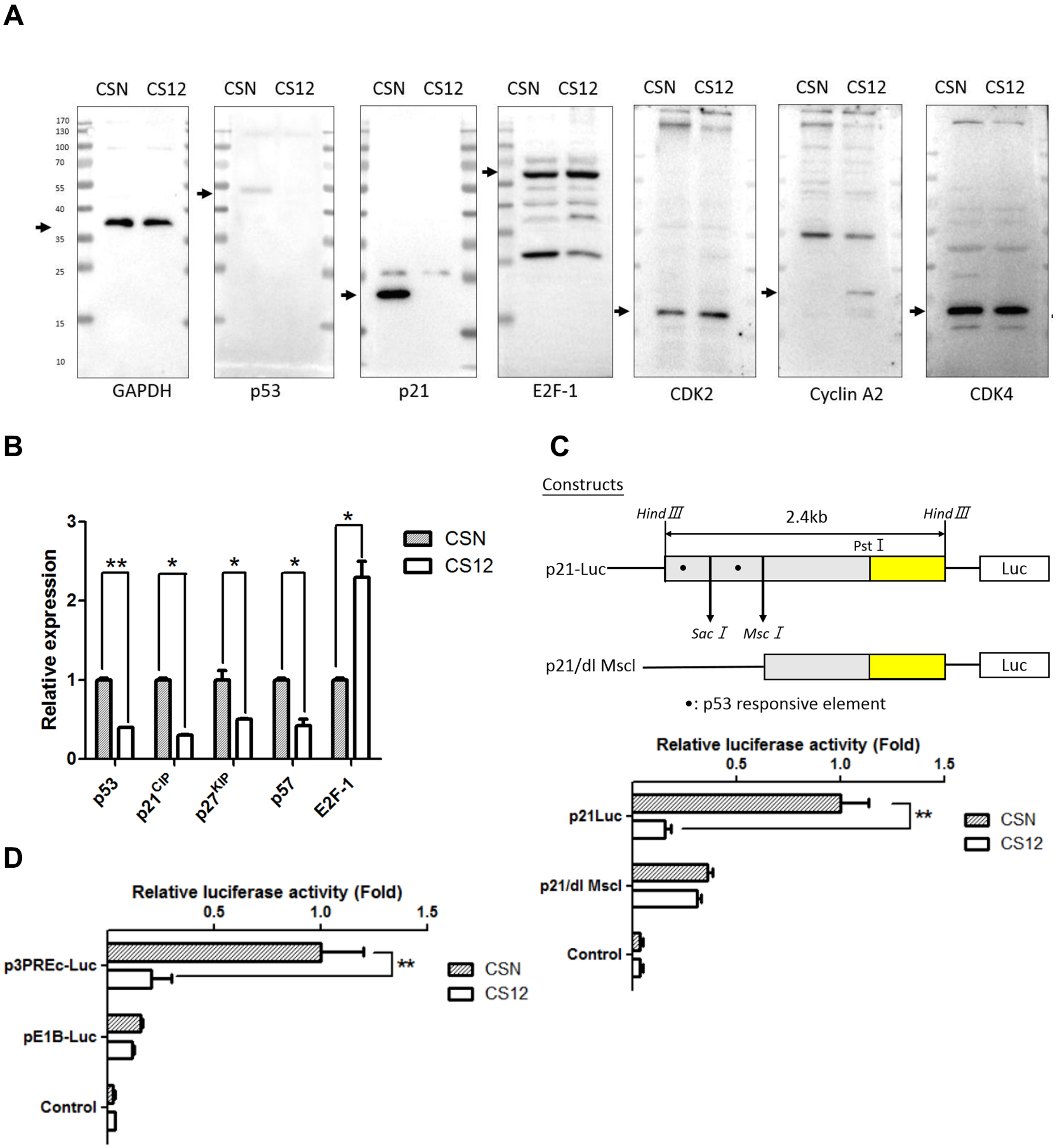

Supplementary Figure S4: Comparative expression of cell cycle regulators in CSN and CS12 cells. (**A**) Comparative expression levels of cell cycle related proteins were examined by Western blot as described in Materials and Methods. Arrows showed the indicated proteins. (**B**) Relative RNA expression levels of cell cycle related genes. Data were derived from three independent experiments and are presented as mean ± SEM (Two tailed Student's *t* test; $^{**}P < 0.01$). (**C**) Comparison of activation of the p21^{Cip1} promoter in CSN cells and CS12 cells. Schematic representation of p2^{1Cip1} promoter constructs. p21-Luc, WT p21^{Cip1} promoter; p21/dlMsc1, mutant p21^{Cip1} promoter with deletion of upstream and downstream p53 response elements. Activation of the p21^{Cip1} promoter in CSN cells and CS12 cells. Four hundred ng of p21^{Cip1}, the promoter plasmid, was transfected into CSN cells and CS12 cells, and the luciferase activity was measured. The values are expressed as means ± S.D. and *t* tests were used to compare these values with the results for p21-Luc in CSN cells set as 1.0 ($n = 5$, $^{**}P < 0.01$). (**D**) The luciferase activity obtained after transfection with p3PREc-Luc (three copies of consensus p53 response elements) was calculated relative to that of pE1B-Luc (control reporter with minimal E1B TATA). The luciferase activities of p3PREc-Luc was set as 1.0. ($n = 5$, $^{**}P < 0.01$).

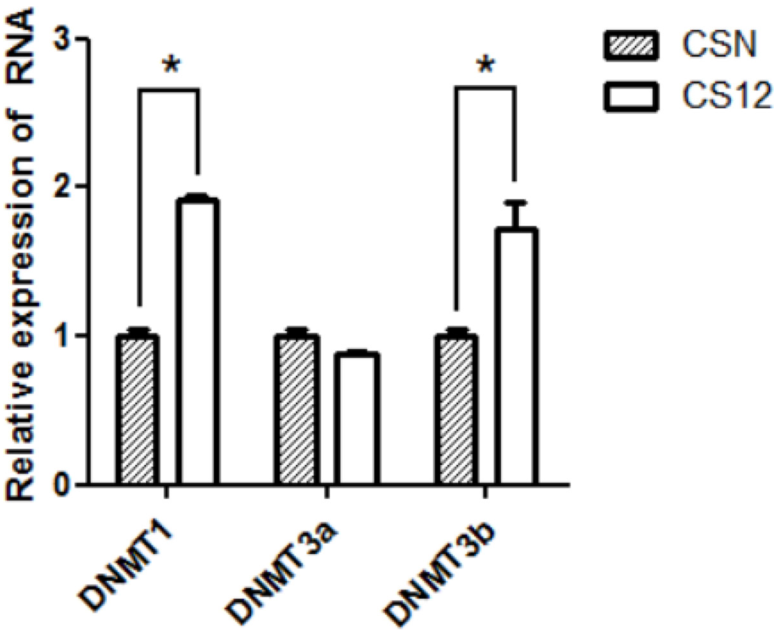

Supplementary Figure S5: qPCR analysis of DNA methylation enzyme genes. Comparative expression of DNMT1, DNMT3a and DNMT3b was examined by qPCR study as described in Materials and Methods. Data were derived from three independent experiments and are presented as mean ± SEM (Two tailed Student's *t* test; *$P < 0.05$).

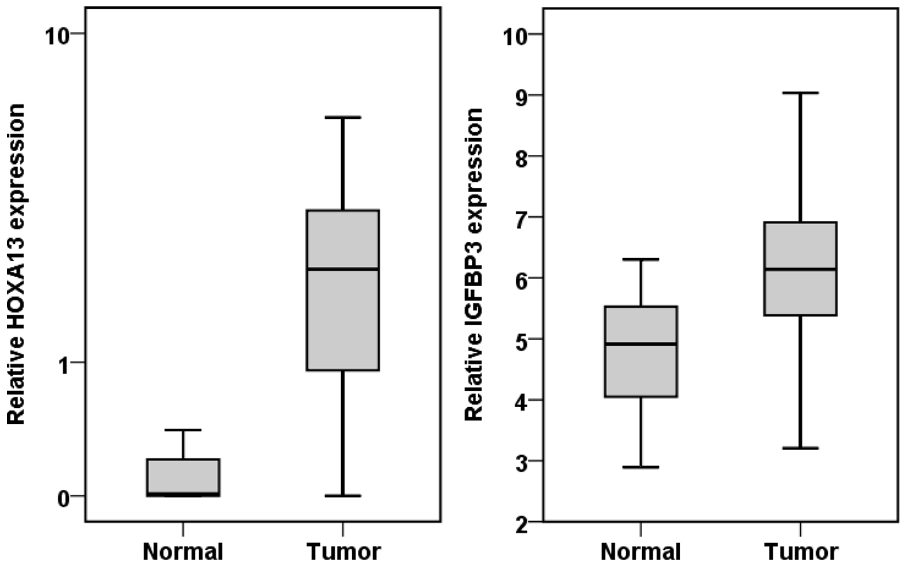

Supplementary Figure S6: Relative expression levels of mRNAs encoding HoxA13 and IGFBP-3 in gastric cancer. The data were retrieved from The Cancer Genome Atlas (version: 2015–02-24) and shown as box plots. The analyzed samples included 37 normal and 384 tumor tissues.

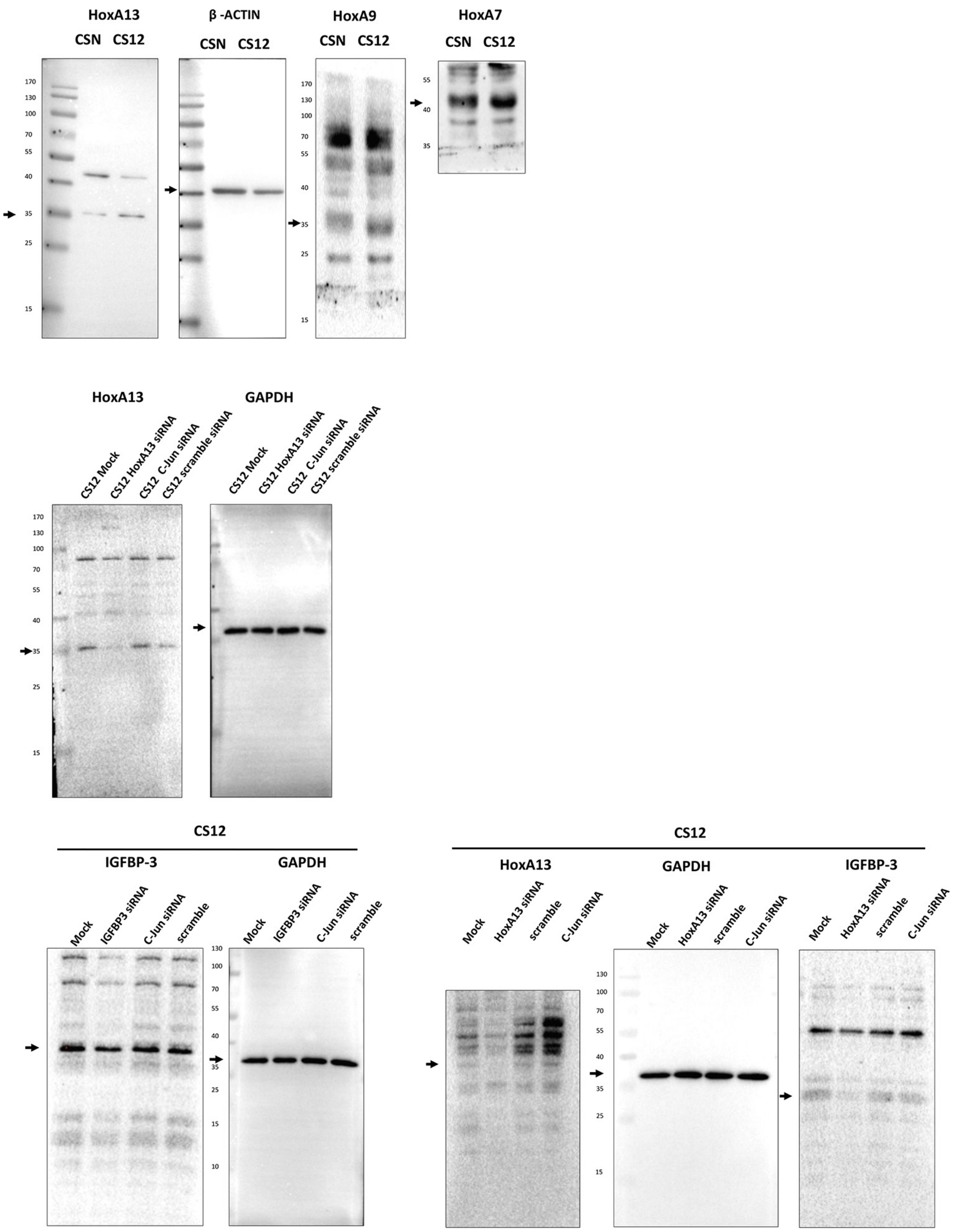

Supplementary Figure S7: Full length western blots used in this work. The bar boxes are as indicated in respective Figures. The molecular weight markers and the protein bands were indicated (arrowheads).

Supplementary Table S1: HOXA13 and IGFBP1 expression in gastric cancer

	HOXA13 ($N = 57$)		IGFBP3 ($N = 28$)	
Positivity	42	73.7%	14	50.0%
Score[a] ≥ 4	36	63.2%	8	28.6%

[a]IHC score: 0 (no expression) to 7 (high expression).

Supplementary Table S2: A list of antibodies used in this work

Antibodies against	Cat. No.	Companies
OCT4	2750	Cell Signaling Technology
SOX2	39844	Active Motif
KLF4	AF3640	R & D System
c-MYC	4609	Cell Signaling Technology
	ab32	Abcam
	ab56	Abcam
NANOG	61420	Active motif
	Ab21624	Abcam
LGR 5	UM370104	OriGene Tech.
CD44variant	MAB12149	Abnova corp.
MMP2	4022	Cell Signaling Technology
MMP9	2270	Cell Signaling Technology
SNAIL	AF3639	R & D System
TWIST	ab50887	Abcam
ZEB1	sc-25388x	Santa Cruz Biotech Inc.
	61120	Active Motif
Cdk2	78B2	Cell Signaling Technology
Cdk4	12790	Cell Signaling Technology
Cyclin A2	sc-239x	Santa Cruz Biotech Inc.
SSEA-1	09-0005	Stegment®
SSEA-3	09-0014	Stegment®
SSEA-4	09-0006	Stegment®
SSEA-5	MA1-144	Thermo Fischer Scientific
Tra-1-60	MAB4360A4	Millipore
Tra-1-81	MAB4381A4	Millipore
Tra-1-85	FCMAB248A4	Millipore
p53	9282	Cell Signaling Technology
	SAB5100112	Sigma Aldrich
RB-1	sc-50x	Santa Cruz Biotech Inc.
	9309	Cell Signaling Technology
p107	sc-318x	Santa Cruz Biotech Inc.
p130	sc-317x	Santa Cruz Biotech Inc.
$p21^{Cip1}$	sc-377x	Santa Cruz Biotech Inc.
	MAB1047	R & D System
$p27^{Kip1}$	2552	Cell Signaling Technology
p57	2557	Cell Signaling Technology
E-Cadherin	610404	BD Biosciences
C-Jun	60A8	Cell Signaling Technology

Brd4	Sc-48872x	Santa Cruz Biotech Inc.
	39910	Active Motif
AFP	AJ1019	Abgent Inc.
ABCB1	AP6111a	Abgent Inc.
CCND3	sc-182	Santa Cruz Biotech Inc.
JNK	sc-571	Santa Cruz Biotech Inc.
pJNK	81E11	Cell Signaling Technology
p-C-Jun (Ser63)	9164	Cell Signaling Technology
p-C-Jun (Ser73)	D47G9	Cell Signaling Technology
GFP	ab290	Abcam
Pol II	ab5408	Abcam
HoxA1	sc-17146x	Santa Cruz Biotech Inc.
HoxA2	sc-17149x	Santa Cruz Biotech Inc.
HoxA4	sc-82886x	Santa Cruz Biotech Inc.
HoxA5	sc-28593x	Santa Cruz Biotech Inc.
HoxA7	sc-17155x	Santa Cruz Biotech Inc.
	09-086	Millipore
HoxA9	07-178	Upstate
HoxA10	sc-17158	Santa Cruz Biotech Inc.
HoxA11	sc-48542x	Santa Cruz Biotech Inc.
HoxA13	sc-46123x	Santa Cruz Biotech Inc.
	Ab26084	Abcam
HoxC13	sc-82906x	Santa Cruz Biotech Inc.
HoxD13	sc-46364x	Santa Cruz Biotech Inc.
H3K4me3	ab8580	Abcam
	61379	Active Motif
H3K27me3	39158	Active Motif
	39157	Active Motif
	SAB4000015	Sigma Aldrich
H3K27me2	61435	Active Motif
H3K9me3	39766	Active Motif
	SAB4000004	Sigma Aldrich
H3K9me2	39754	Active Motif
DNMT1	61468	Active Motif
DNMT3a	39206	Active Motif
DNMT3b	39207	Active Motif
DNMT3L<br>WDR5	A-1005050	Epigenetic Group Inc.
	ab56919	Abcam
	A302-429A	Bethyl Lab. Inc.
MLL1	A300-087A	Bethyl Lab. Inc.
SUZ12	3737	Cell Signaling Technology
TET1	61444	Active Motif
TET2	MBP2-43552	Novus Biologicals
IGFBP-3	orb10884	Biorbyt
	ab36529	Abcam
	MA1-2785	Thermo Fischer Scientific
	sc-9028	Santa Cruz Biotech Inc.

β-Actin	sc81178	Santa Cruz Biotech Inc.
	4967	Cell Signaling Technology
	ab8227	Abcam
	ab6276	Abcam
GAPDH	MAB374	Millipore
	2118	Cell Signaling Technology
	ab8245	Abcam
	ab9485	Abcam

Supplementary Table S3: Primer sequences of stemness genes for RT-PCR

Name of genes	Sequences of primers
OCT3/4	Fw: 5′-GGGTTTTTGGGATTAAGTTCTTCA-3′<br>Rw: 5′-GCCCCCACCCTTGTGTT-3′
SOX2	Fw: 5′-CAAAAATGGCCATGCAGGTT-3′<br>Rw: 5′-AGTTGGGATCGAACAAAAGCTATT-3′
KLF4	Fw: 5′-ACCTAAATGATGGTGCTTGCT-3′<br>Rw: 5′-TTGAAAACTTTGGCTTCCTTGTT-3′
C-MYC	Fw: 5′-CGGGCGGGCACTTTG-3′<br>Rw: 5′-GGAGAGTCGCGTCCTTGCT-3′
NANOG	Fw: 5′-CAGTCTGGACACTGGCTGAA-3′<br>Rw: 5′-CTCGTGATTAGGCTCCAAC-3′
hTERT	Fw: 5′-AGAGTGTCTGGAGCAAGTTGC-3′<br>Rw: 5′-CGTAGTCCATCCATGTTCAATCG-3′
REX	Fw: 5′-CAGATCCTAAACAGCTCGCAGAAT-3′<br>Rw: 5′-GCGTACGCAAATTAAAGTCCGA-3′
JDP2	Fw: 5′-GATGCCGGAACAAGAAGAA-3′<br>Fw: 5′-GCTTCAGCTCCTCAATCTGG-3′
GAPDH	Fw: 5′-AGGAGCGAGATCCCTCCAAA-3′<br>Rw: 5′-TGATGACCCTTTTGGCTCCC-3′
β-ACTIN	Fw: 5′-AGGCACCAGGGCGTGAT-3′<br>Rw: 5′-GCCCACATAGGAATCCTTCTGAC-3′

Supplementary Table S4: Primer sequences of Hox family for RT-PCR

Name of genes	Sequences of primers
HoxA1	Fw: 5′-CTTCTCCAGCGCAGACTTTT-3′<br>Rw: 5′-CGCGTCAGGTACTTGTTGAA-3′
HoxA2	Fw: 5′-CCACAAAGAATCCCTGGAAA-3′<br>Rw: 5′-CCGGTTCTGAAACCACACTT-3′
HoxA3	Fw: 5′-CACAAAGCAGAAAACCGGCA-3′<br>Rw: 5′-ACAGGTAGCGGTTGAAGTGG-3′
HoxA4	Fw: 5′-CAAGGAGCCCGTGGTGTA-3′<br>Rw: 5′-CAGACAAACAGAGCGTGTGG-3′
HoxA5	Fw: 5′-CCCAGATCTACCCCTGGATG-3′<br>Rw: 5′-GGAAGGCAAAGAGCATGTG-3′
HoxA7	Fw: 5′-TATAATTTTGATTTGTGATTGTTGTT-3′<br>Rw: 5′-AAACCTCTTACCCTTCCATTCTAAA-3′
HoxA9	Fw: 5′-CTCAGGAGCCTCGTGTCTTT-3′<br>Rw: 5′-CAGTTCCAGGGTCTGGTGTT-3′
HoxA10	Fw: 5′-CGCTCCCCTTCATAACAGAA-3′<br>Fw: 5′-TGCGTTTTCACCTTTGGAAT-3′
HoxA13	Fw: 5′-CCTCTGGAAGTCCACTCTGC-3′<br>Rw: 5′-GGTATAAGGCACGCGCTTC-3′

HoxC13	Fw: 5′-TCAGGTGTACTGCTCCAAGG-3′<br>Rw: 5′-CAGCTGCACCTTAGTGTAGGG-3′
HoxD13	Fw: 5′-AGAATGGAGAACGAGTATGCC-3′<br>Rw: 5′-CGGTTCTGAAACCAAATGC-3′
GAPDH	Fw: 5′-ACCCACTCCTCCACCTTTGAC-3′<br>Rw: 5′-TGTTGCTGTAGCCAAATTCGTT-3′

Supplementary Table S5: Primer sequences of quantitative real-time RT-PCR

Name of genes	Sequences of primers
HoxA1	Fw: 5′-ACATCTTCTCCAGCGCAGAC-3′<br>Rw: 5′-CGTGAGCTGCTTGGAGTGA-3′
HoxA4	Fw: 5′-TCCCCATCTGGACCATAATAGG-3′<br>Rw: 5′-GCAACCAGCACAGACTCTTAACC-3′
HoxA7	Fw: 5′-AAAGCGCGTTCACATAATAC-3′<br>Rw: 5′-GTTATCATATATCACTCTACCTCGT-3′
HoxA9	Fw: 5′-AGGAGTCGCTGCTTTCTGTT-3′<br>Rw: 5′-ATTAGAACGGGAGGGGTAA-3′
HoxA11	Fw: 5′-GTACTTACTACGTCTCGGGTCCAG-3′<br>Rw: 5′-AGTCTCTGTGCACGAGCTCCT-3′
HoxA13	Fw: 5′-AAATGTACTGCCCCAAAGAGCA-3′<br>Rw: 5′-ATCCGAGGGATGGGAGACC-3′
HoxC13	Fw: 5′-AAGGTGGTCAGCAAATCGAAG-3′<br>Rw: 5′-TGGTACAAAGCGGAGACATAAATAGA-3′
HoxD13	Fw: 5′-CTGGGCTACGGCTACCACTTC-3′<br>Fw: 5′-GCGATGACTTGAGCGCATT-3′
GAPDH	Fw: 5′-GATTCCACCCATGCAAATCC-3′<br>Rw: 5′-TGGGATTTCCATTGATGACAAG-3′
HoxC13	Fw: 5′-TCAGGTGTACTGCTCCAAGG-3′<br>Rw: 5′-CAGCTGCACCTTAGTGTAGGG-3′
HoxD13	Fw: 5′-AGAATGGAGAACGAGTATGCC-3′<br>Rw: 5′-CGGTTCTGAAACCAAATGC-3′
GAPDH	Fw: 5′-ACCCACTCCTCCACCTTTGAC-3′<br>Rw: 5′-TGTTGCTGTAGCCAAATTCGTT-3′

Supplementary Table S6: Primer sequences of various genes for qPCR

Name of genes	Sequences of primers
HOTTIP	Fw: 5′-CCTAAAGCCACGCTTCTTTG-3′<br>Rw: 5′-TGCAGGCTGGAGATCCTACT-3′
H19	Fw: 5′-TGCTGCACTTTACAACCACTG-3′<br>Rw: 5′-ATGGTGTCTTTGATGTTGGGC-3′
HOTAIRM1	Fw: 5′-TTGGAGTGCTGGAGCGAA-3′<br>Rw: 5′-TCCTCATTGCCATGCTAATGACGAGAGG-3′
HOTAIR	Fw: 5′-GGTAGAAAAAGCAACCACGAAGC-3′<br>Rw: 5′-ACATAAACCTCTGTTCTGTGAGTGCC-3′
E2F1	Fw: 5′-AAGAGCAAACAAGGCCCGAT-3′<br>Rw: 5′-tggtggtggtgacactatgg-3′
p53	Fw: 5′-TGAGCGCTTCGAGATGTTCC-3′<br>Rw: 5′-TTTGGACTTCAGGTGGCTGG-3′
$p21^{CIP}$	Fw: 5′-ATGTCCGTCAGAACCCATGC-3′<br>Rw: 5′-TCGAAGTTCCATCGCTCACG-3′
$p27^{KIP}$	Fw: 5′-GAGCAATGCGCGCAGGAATAAGG-3′<br>Rw: 5′-TTGGGAACCGTCTGAAACA-3′

p57	Fw: 5′-ATCCACGATGGAGCGTCTTG-3′<br>Rw: 5′-TCGTAATCCCAGCGGTTCTG-3′
RB1	Fw: 5′-TCTCACCTCCCATGTTGCTC-3′<br>Rw: 5′-GCTATCCGTGCACTCCTGTT-3′
DNMT1	Fw: 5′-CCTTGGAGAACGGTGCTCAT-3′<br>Rw: 5′-GCATCTGCCATTCCCACTCT-3′
DNMT3A	Fw: 5′-CCCAGGCAGCCATTAAGGA-3′<br>Rw: 5′-AGCGAAGAACATCTGGAGCC-3′
DNMT3B	Fw: 5′-CCCAGGCAGCCATTAAGGAA-3′<br>Rw: 5′-TGGGCTTCTGAACGAGTCC-3′
IGFBP-3	Fw: 5′-AATGGCCGCGGGTTCTGC-3′<br>Rw: 5′-TTCTGGGTGTCTGTGCTTTGAG-3′
GAPDH	Fw: 5′-AGGAGCGAGATCCCTCCAAA-3′<br>Rw: 5′-TGATGACCCTTTTGGCTCCC-3′

Supplementary Table S7: Primer sequences of ChIP assay

Name of genes	Sequences of primers
E1	Fw: 5′-GCTTTGCATACGCCGTGGG -3′<br>Rw: 5′-CCAAGAAGTGAAGAGCGC-3′
NS	Fw: 5′-ACCCATTCTGAGCTAGGCT-3′<br>Rw: 5′-ATCTGGAGCAGTGGGGACA-3′
